# Physiologically Based Pharmacokinetic Modeling of Metoprolol Enantiomers and α-Hydroxymetoprolol to Describe CYP2D6 Drug-Gene Interactions

**DOI:** 10.3390/pharmaceutics12121200

**Published:** 2020-12-11

**Authors:** Simeon Rüdesheim, Jan-Georg Wojtyniak, Dominik Selzer, Nina Hanke, Felix Mahfoud, Matthias Schwab, Thorsten Lehr

**Affiliations:** 1 Clinical Pharmacy, Saarland University, 66123 Saarbrücken, Germany; simeon.ruedesheim@uni-saarland.de (S.R.); jangeorg.wojtyniak@uni-saarland.de (J.-G.W.); dominik.selzer@uni-saarland.de (D.S.); n.hanke@mx.uni-saarland.de (N.H.); 2 Dr. Margarete Fischer-Bosch—Institute of Clinical Pharmacology, 70376 Stuttgart, Germany; matthias.schwab@ikp-stuttgart.de; 3 Department of Internal Medicine III, Cardiology, Angiology, Intensive Care Medicine, Saarland University Medical Center and Saarland University Faculty of Medicine, 66421 Homburg, Germany; felix.mahfoud@uks.eu; 4 Institute for Medical Engineering and Science, Massachusetts Institute of Technology, Cambridge, MA 02139, USA; 5 Departments of Clinical Pharmacology, Pharmacy and Biochemistry, University of Tübingen, 72076 Tübingen, Germany; 6 Cluster of Excellence iFIT (EXC2180) “Image-Guided and Functionally Instructed Tumor Therapies”, University of Tübingen, 72076 Tübingen, Germany

**Keywords:** physiologically based pharmacokinetic (PBPK) modeling, metoprolol, metoprolol enantiomers, α-hydroxymetoprolol, drug-gene interactions (DGIs), cytochrome P450 2D6 (CYP2D6), dose adaptation, model-informed precision dosing

## Abstract

The beta-blocker metoprolol (the sixth most commonly prescribed drug in the USA in 2017) is subject to considerable drug–gene interaction (DGI) effects caused by genetic variations of the *CYP2D6* gene. CYP2D6 poor metabolizers (5.7% of US population) show approximately five-fold higher metoprolol exposure compared to CYP2D6 normal metabolizers. This study aimed to develop a whole-body physiologically based pharmacokinetic (PBPK) model to predict CYP2D6 DGIs with metoprolol. The metoprolol (*R*)- and (*S*)-enantiomers as well as the active metabolite α-hydroxymetoprolol were implemented as model compounds, employing data of 48 different clinical studies (dosing range 5–200 mg). To mechanistically describe the effect of CYP2D6 polymorphisms, two separate metabolic CYP2D6 pathways (α-hydroxylation and *O*-demethylation) were incorporated for both metoprolol enantiomers. The good model performance is demonstrated in predicted plasma concentration–time profiles compared to observed data, goodness-of-fit plots, and low geometric mean fold errors of the predicted AUC_last_ (1.27) and C_max_ values (1.23) over all studies. For DGI predictions, 18 out of 18 DGI AUC_last_ ratios and 18 out of 18 DGI C_max_ ratios were within two-fold of the observed ratios. The newly developed and carefully validated model was applied to calculate dose recommendations for CYP2D6 polymorphic patients and will be freely available in the Open Systems Pharmacology repository.

## 1. Introduction 

Metoprolol is one of the most frequently administered beta-blockers in the U.S. with well over 50 million total prescriptions per year [1]. It is used in the treatment of hypertension, coronary artery disease, heart failure, and arterial fibrillation [2]. Metoprolol is listed by the U.S. Food and Drug Administration (FDA) as a moderately sensitive substrate for clinical drug-drug interaction (DDI) studies as it is predominantly metabolized by cytochrome P450 2D6 (CYP2D6) [3]. 

CYP2D6 is an important drug metabolizing enzyme which is estimated to contribute to the metabolism of 15–25% of all clinically used drugs [4,5]. The gene encoding CYP2D6 is subject to different genetic variations, ranging from null alleles to several-fold amplification [5], resulting in considerable phenotypical interindividual differences in CYP2D6-dependent drug metabolism [6]. The main purpose of the CYP2D6 activity score (AS) is to translate a patients’ *CYP2D6* genotype to the corresponding phenotype [7]. For this, *CYP2D6* alleles are assigned a value indicating no (0), decreased (0.25 or 0.5), normal function (1), or a copy number variation of a normal function allele (2). However, as this assignment is based on semiquantitative observations, an activity score of 0.5 does not necessarily imply a reduction of enzymatic activity by 50% [6,8]. Nevertheless, the activity score has been shown to correlate well with metoprolol oral clearance in vivo [9]. Yet, considerable interindividual variability in metoprolol plasma concentrations, caused by genetic components independent of the *CYP2D6* genotype, such as the rs5758550 SNP, has been observed [9,10].

Metoprolol is a BCS Class I drug, characterized by high permeability and high solubility. After its rapid absorption, metoprolol undergoes extensive first-pass metabolism, reducing its bioavailability to 40% in CYP2D6 normal metabolizers (NMs), whereas bioavailability approaches 100% in poor metabolizers (PMs) [11]. Only 12% of metoprolol are bound to plasma proteins, primarily albumin [12]. *O*-demethylation, α-hydroxylation, and *N*-dealkylation by CYP2D6 and, to lesser extents, CYP2B6, CYP2C9, and CYP3A4, are described as the pathways of metoprolol metabolism [13,14]. Of the major metabolites, α-hydroxymetoprolol is of particular clinical interest, as it is pharmacologically active, exhibiting 10% of the β_1_-blocking activity of metoprolol [15], and it is almost exclusively formed via CYP2D6 [16]. Therefore, α-hydroxymetoprolol/metoprolol urinary metabolic ratios are employed for CYP2D6 phenotyping [17]. Overall, CYP2D6 is estimated to be responsible for 80% of metoprolol metabolism in normal metabolizers [14]. Depending on the CYP2D6 phenotype, only 1.5–12% of orally administered metoprolol are excreted unchanged in urine [18]. 

Metoprolol is a chiral molecule, marketed as a racemic mixture of *(R)*- and *(S)*-metoprolol, even though its enantiomers differ in their pharmacodynamic and pharmacokinetic properties. The (*S*)-enantiomer has been shown to be 33-fold more potent in blocking β_1_-adrenoceptors in rats than the (*R*)-enantiomer [19]. Moreover, in ultrarapid metabolizers (UMs) and normal metabolizers, but not in poor metabolizers, the (*S*)-metoprolol area under the plasma concentration–time curve (AUC) is significantly higher than the AUC of *(R)*-metoprolol, showing the enantiopreference of CYP2D6 towards the (*R*)-enantiomer [18,20]. The distribution of *CYP2D6* genotypes varies substantially between ethnicities. For instance, 5.7% of the US and 0.9% of Middle Eastern or Oceanian populations were found to be poor metabolizers (AS = 0), whereas the prevalence of ultrarapid metabolizers (AS > 2) was 2.2% in the US and 11.2% in Middle Eastern or Oceanian populations [21,22]. Interestingly, the reduced-function CYP2D6*10 allele occurs more often in East Asian populations than the CYP2D6*1 allele (42% vs. 34%), which results in an overall decreased CYP2D6 activity compared to other populations [23].

Previously published metoprolol PBPK models were either based on traditional CYP2D6 phenotypes [24,25] or did not take CYP2D6 DGIs into consideration [26,27]. Moreover, none of the previously published metoprolol PBPK models incorporated the metoprolol (*R*)- and (*S*)-enantiomers to describe the enantioselective metabolism via CYP2D6.

This study aimed to develop and qualify a novel, whole-body physiologically based pharmacokinetic (PBPK) model of metoprolol to describe the effects of the different *CYP2D6* genotypes and the resulting activity scores on the pharmacokinetics of metoprolol. The resulting drug–gene interaction (DGI) PBPK model includes (*R*)- and *(S*)-metoprolol with their specific CYP2D6 activity score-dependent metabolism, as well as the metabolite α-hydroxymetoprolol. In addition, the established model was applied to generate metoprolol dose adaptations for patients with different CYP2D6 activity scores and these adaptations were compared to a current guideline [28]. The model was developed as a whole-body PBPK model to allow future model applications such as DDI modeling, model scaling to special populations or PBPK-PD modeling. The final PBPK model will be publicly available in the Open Systems Pharmacology (OSP) repository (www.open-systems-pharmacology.org) [29] as a clinical research tool, and the Supplementary Materials to this article provide a detailed and transparent evaluation of the model performance to be used as a reference manual and evaluation report. 

## 2. Materials and Methods 

### 2.1. Software 

PBPK modeling, model parameter optimization (Monte Carlo algorithm), and local sensitivity analysis were performed using PK-Sim^®^ and MoBi^®^ (Open Systems Pharmacology Suite 9.1). Published clinical study data were digitized with GetData Graph Digitizer 2.26.0.20 (© S. Fedorov) according to best practices [30]. Pharmacokinetic parameters (area under the plasma concentration-time curve from the time of the first concentration measurement to the time of the last concentration measurement (AUC_last_) and maximum plasma concentration (C_max_)) and model performance metrics (mean relative deviation (MRD), geometric mean fold error (GMFE), DGI AUC_last_, and C_max_ ratios) were calculated using Python (version 3.7.4, Python Software Foundation, Wilmington, DE, USA) in Visual Studio Code (version 1.49.1, Microsoft Corporation, Redmond, WA, USA). Plots were also generated using Python in Visual Studio Code.

### 2.2. PBPK Model Building

The PBPK model building was initiated with an extensive literature search to gather information on metoprolol absorption, distribution, metabolism, and excretion (ADME) processes, to obtain physicochemical data and to collect clinical studies of the intravenous and oral administration of metoprolol, in single- and multiple-dose regimens, performed in healthy individuals. Subsequently, plasma concentration-time profiles from the published clinical studies were digitized and split into a training dataset, for model building, and a test dataset, for model evaluation. Studies for model training were selected to include different routes of administration (intravenous and oral), a wide range of administered doses, single- and multiple-dose regimens, as well as stratification for *CYP2D6* genotype or activity score. The training dataset was used for estimation of model input parameters which could not be obtained from literature. 

**Figure 1:**
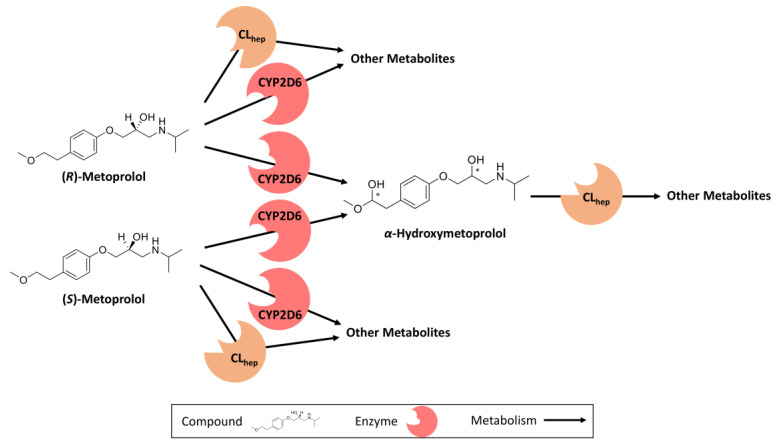
Implemented metoprolol metabolic pathways. (*R*)- and (*S*)-metoprolol are both metabolized via two different CYP2D6-dependent metabolic pathways: α-hydroxylation and *O*-demethylation, as well as by an unspecific hepatic clearance process. The four α-hydroxymetoprolol diastereomers (stereocenters are marked with asterisks) were modeled as one single compound due to lacking published clinical data. CL_hep_: hepatic clearance, CYP2D6: cytochrome P450 2D6.

The metoprolol PBPK model was built in a stepwise approach. First, appropriate quantitative structure-activity relationship (QSAR) methods to estimate the cellular permeabilities and partition coefficients (e.g., Rodgers & Rowland, Berezhkovskiy) were selected, by fitting simulations of intravenous metoprolol administration to their observed data. Subsequently, studies of orally administered metoprolol in poor metabolizers were used to optimize parameters independent of CYP2D6 metabolism. A single study in which metoprolol was administered as an oral solution was used to optimize the intestinal permeability for both metoprolol enantiomers [31]. Finally, *(R)*- and *(S)*-enantiomer CYP2D6 catalytic rate constant (k_cat_) values were optimized for studies of the training dataset where the volunteers were either normal metabolizers or not phenotyped. Racemic metoprolol plasma concentration–time profiles were modeled by the administration of racemic doses of metoprolol (50% (*R*)- and 50% (*S*)-metoprolol and the use of a customized “observer” within PK-Sim^®^, which adds up the simulated (*R*)- and (*S*)-metoprolol plasma concentrations to directly display the racemic metoprolol plasma concentration–time profiles. Figure 1 provides an overview of metoprolol metabolic pathways.

Supplementary Table S2.2.1 contains information concerning all studies included in the training and test datasets. Supplementary Table S4.0.1 provides system-dependent parameters with technical details on the implementation of CYP2D6. 

### 2.3. DGI Modeling 

The metoprolol clearance processes via CYP2D6 were implemented using Michaelis–Menten kinetics according to Equation (1) [32]:

(1) $$v\;=\;\frac{v_{\max\;}\cdot \;S}{K_{m}\;+\;S}\;=\;\frac{k_{\mathrm{cat}}\;\cdot \;E\;\cdot \;S}{K_{m}\;+\;S}$$

where v = reaction velocity, v_max_ = maximum reaction velocity, S = free substrate concentration, K_m_ = Michaelis-Menten constant, k_cat_ = catalytic rate constant, and E = enzyme concentration.

CYP2D6 Michaelis–Menten constant (K_m_) values were kept constant over the whole range of modeled activity scores. CYP2D6 k_cat_ values were optimized for each activity score separately. CYP2D6 poor metabolizers (AS = 0) were assumed to show no CYP2D6 activity (0%), whereas populations with two wildtype alleles (AS = 2) were used as reference (100%) to calculate relative k_cat_ values according to Equation (2).

(2) $$k_{\mathrm{cat},\;\mathrm{rel},\;\mathrm{AS}=i}\;=\;\frac{k_{\mathrm{cat},\;\mathrm{AS}\;=\;i}}{k_{\mathrm{cat},\;\mathrm{AS}\;=\;2}}\times 100\%$$

where k_cat, rel, AS=i_ = k_cat_ for the investigated activity score relative to AS = 2, k_cat, AS=i_ = k_cat_ for the investigated activity score, and k_cat, AS = 2_ = k_cat_ for AS = 2.

The assignment of activity scores was carried out according to [33] as described in Table 1.

### 2.4. PBPK Model Evaluation 

The performance of the metoprolol PBPK model regarding the prediction of racemic metoprolol, its enantiomers and α-hydroxymetoprolol was evaluated using graphical and statistical methods. First, predicted plasma concentration-time profiles were compared graphically with the profiles measured in the respective clinical studies by plotting model population predictions (arithmetic mean ± SD) together with observed data points. For this purpose, virtual populations of 100 individuals were created based on the population characteristics stated in the respective publication. System-dependent parameters, such as age, weight, height, organ weights, blood flow rates, tissue composition, etc., were varied by the implemented algorithm in PK-Sim. A comprehensive description of virtual populations is given in Supplementary Section S1.1.3. Second, the plasma concentration values of all studies predicted using the arithmetic mean of the population were plotted against their corresponding observed values in goodness-of-fit plots. 

In addition, model performance was evaluated by a comparison of predicted to observed AUC values and C_max_ values. All AUC values (predicted as well as observed) were calculated from the time of the first concentration measurement to the time of the last concentration measurement (AUC_last_). 

As quantitative measures of the model performance, the MRD of all predicted plasma concentrations (Equation (3)) and the GMFE of all predicted AUC_last_ and C_max_ values (Equation (4)) were calculated.

(3) $${\mathrm{MRD}\;=\;10}^{x};\;x\;=\;\sqrt{\frac{\sum_{i=1}^{k}{\;(\log}_{10}\hat{c_{i}}{\;-\;\log}_{10}c_{i}{)}^{2}}{k}}$$

where $\hat{c_{i}}$ = predicted plasma concentration that corresponds to the *i*-th observed concentration, $c_{i}$ = *i*-the observed plasma concentration, and k = number of observed values.

(4) $${\mathrm{GMFE}\;=\;10}^{x};\;x\;=\;\frac{\sum_{i=1}^{m}\left|\log_{10}\;\left(\frac{\hat{\rho_{i}}}{\rho_{i}}\right)\right|\;}{m}$$

where $\hat{\rho_{i}}$ = predicted AUC_last_ or C_max_ value of study i, $\rho_{i}$ = corresponding observed AUC_last_ or C_max_ value of study i, and m = number of studies. 

A detailed description of the local sensitivity analysis is provided in Supplementary Section S1.2.2.

### 2.5. DGI Modeling Evaluation 

The DGI modeling performance was assessed by a comparison of predicted versus observed plasma concentration–time profiles of racemic metoprolol, its enantiomers, and α-hydroxymetoprolol. Furthermore, predicted DGI AUC_last_ ratios (Equation (5)) and DGI C_max_ ratios (Equation (6)) were evaluated to assess, if the impact of the observed DGIs was well described by the model.

(5) $$\mathrm{DGI}\;{\mathrm{AUC}}_{\mathrm{last}\;}\;\mathrm{ratio}\;=\;\frac{{\mathrm{AUC}}_{\mathrm{last},\;\mathrm{DGI}}\;}{{\mathrm{AUC}}_{\mathrm{last},\;\mathrm{reference}}\;}$$

where AUC_last, DGI_ = AUC_last_ of variant activity score or phenotype, while AUC_last, reference_ = AUC_last_ of AS = 2 or normal metabolizer phenotype.

(6) $$\mathrm{DGI}\;C_{\max\;}\;\mathrm{ratio}\;=\;\frac{C_{\max,\;\mathrm{DGI}}\;}{C_{\max,\;\mathrm{reference}}\;}$$

where C_max, DGI_ = C_max_ of variant activity score or phenotype, C_max, reference_ = C_max_ of AS = 2 or normal metabolizer phenotype. As a quantitative measure of the prediction accuracy, GMFE values of the predicted DGI AUC_last_ ratios and DGI C_max_ ratios were calculated according to Equation (4). 

## 3. Results 

### 3.1. Metoprolol PBPK Model Development and Evaluation

A total of 48 clinical studies concerning the intravenous or oral administration of metoprolol were used in the model development process, with doses ranging from 5 to 200 mg metoprolol in single or multiple dose regimens. Of the 48 studies, nine included measurements of the metabolite α-hydroxymetoprolol and 16 studies included measurements of the metoprolol enantiomers.

Metoprolol enantiomers were modeled as stand-alone compounds, to allow for the implementation of enantioselective CYP2D6 metabolism. The four α-hydroxymetoprolol diastereomers were modeled as one single compound, due to a lack of enantiomeric differentiation in the published clinical data.

For both metoprolol enantiomers, enantioselective metabolism via CYP2D6, an unspecific hepatic clearance process, as well as passive glomerular filtration were implemented. Each of the metoprolol enantiomers can be metabolized via CYP2D6 to produce either α-hydroxymetoprolol or to generate other metabolites such as *O*-demethylmetoprolol which were not included as separately modeled compounds. The metabolite α-hydroxymetoprolol is eliminated via an unspecific hepatic clearance process. Figure 1 depicts a schematic overview of the implemented metabolic pathways. The drug-dependent model input parameters of the metoprolol enantiomers are presented in Table 2. The drug-dependent parameters of the α-hydroxymetoprolol model are provided in Supplementary Table S2.4.3.

**Figure 2:**
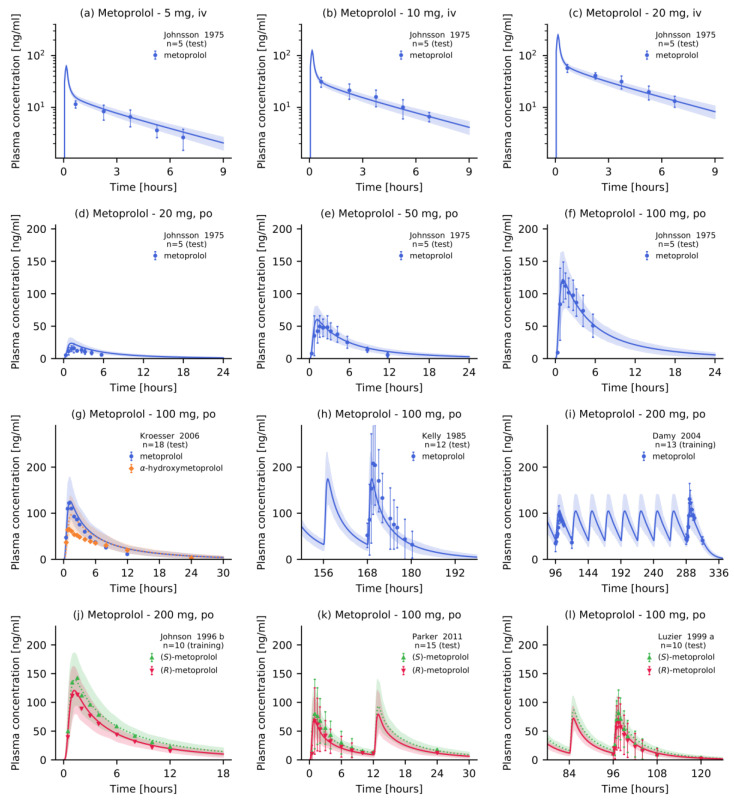
Metoprolol plasma concentrations. Model predictions of metoprolol and its metabolite α-hydroxymetoprolol plasma concentration-time profiles of selected (**a**–**c**) intravenous and (**d**–**l**) oral studies of the training and test datasets, compared to observed data [43,44,45,47,48,49,50]. Population predictions (n = 100) are shown as lines with ribbons (arithmetic mean ± standard deviation (SD)), symbols represent the corresponding observed data ± SD. Detailed information on all clinical studies is listed in Supplementary Table S2.2.1. iv: intravenous, po: oral.

Overall, the PBPK model accurately described and predicted the plasma concentration–time profiles of metoprolol and α-hydroxymetoprolol after intravenous and oral administration, as illustrated in Figure 2. This figure presents population predictions of selected clinical studies from the test and training datasets. Plots documenting the model performance for all 48 clinical studies included in this analysis are provided in Supplementary Sections S2.5 and S3.2. All simulated plasma profiles are in good agreement with the observed metoprolol racemate, (*R*)-, and (*S*)-metoprolol as well as α-hydroxymetoprolol plasma concentrations. 

**Figure 3:**
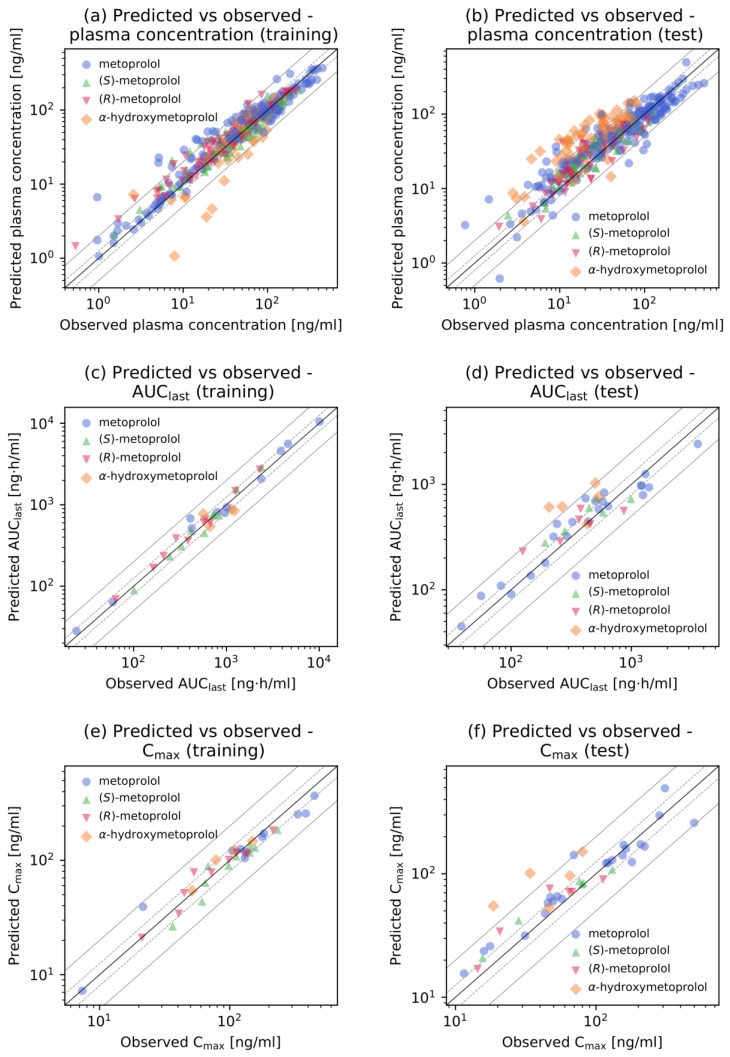
Goodness-of-fit plots of the final metoprolol model. Predicted versus observed (**a**,**b**) plasma concentrations, (**c**,**d**) AUC_last_ values and (**e**,**f**) C_max_ values for the training (left column) and test (right column) datasets. The solid black line indicates the line of identity, solid grey lines show two-fold deviation, dashed grey lines indicate 1.25-fold deviation. Detailed information on all clinical studies is listed in Supplementary Table S2.2.1. AUC_last_: area under the plasma concentration-time curve from the time of the first concentration measurement to the time of the last concentration measurement, C_max_: maximum plasma concentration, vs: versus.

Goodness-of-fit plots showing plasma concentrations, AUC_last_ and C_max_ values, respectively, are presented in Figure 3. Predicted plasma concentrations were predominantly (88.3%) within two-fold of the corresponding observed concentrations. Furthermore, a total of 72 out of 75 of the predicted AUC_last_ values (several studies included measurements of multiple analytes) and 64 out of 66 of the predicted C_max_ values were within the two-fold acceptance criterion. The metoprolol model GMFE values were 1.27 (range 1.01–2.94) for the predicted AUC_last_ values, and 1.23 (range 1.00–2.97) for the predicted C_max_ values. The MRD values and predicted to observed AUC_last_ and C_max_ ratios for all 48 clinical studies and all measured analytes are provided in Supplementary Tables S2.6.4–S2.6.7.

The local sensitivity analysis of a simulation of 100 mg metoprolol tartrate administered orally (standard dose) revealed that the model predictions were most sensitive to the values of (*R*)- and (*S*)-metoprolol fraction unbound (f_u_), which were gathered from literature and used unmodified as model input parameters. Setting a sensitivity threshold of 0.5 (100% parameter value change = 50% change of predicted AUC), the only other parameter value that the model predictions were sensitive to is the CYP2D6 (*R*)-metoprolol → *O*-demethylmetoprolol catalytic rate constant (optimized). A comprehensive visual and quantitative presentation of the sensitivity analysis results can be found in Supplementary Section S2.6.7.

### 3.2. Metoprolol CYP2D6 DGI Model Development and Evaluation

The model training dataset included 11 plasma concentration-time profiles from studies that reported the CYP2D6 activity scores of their study subjects, ranging from 0 (poor metabolizer) to 3 (ultrarapid metabolizer). These studies were utilized to optimize k_cat, rel_ values for the different CYP2D6 activity scores. The identified values for both CYP2D6 pathways and both metoprolol enantiomers are given in Table 3. 

**Figure 4:**
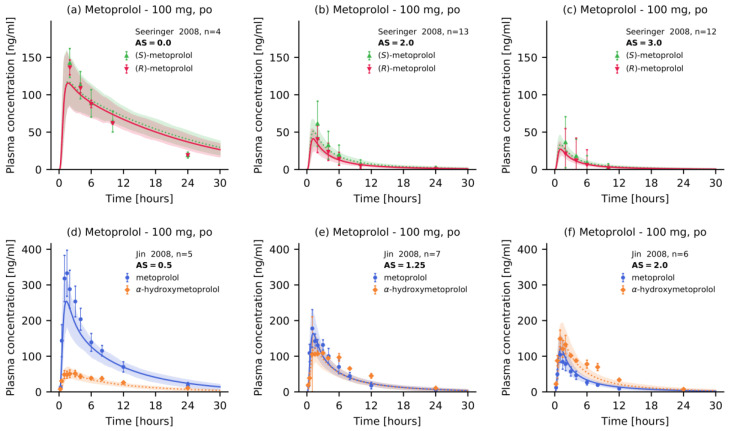
Metoprolol plasma concentrations of the modeled CYP2D6 drug-gene interaction. Model predictions of (**a**–**c**) (R)-metoprolol and (S)-metoprolol as well as (**d**–**f**) metoprolol and α hydroxymetoprolol plasma concentration-time profiles of selected metoprolol CYP2D6 DGI studies, compared to observed data [18,51]. Population predictions (n = 100) are shown as lines with ribbons (arithmetic mean ± standard deviation (SD)), symbols represent the corresponding observed data ± SD. Detailed information on all clinical studies is listed in Supplementary Table S2.2.1. AS: activity score, po: oral.

Of all 48 analyzed clinical profiles, 15 metoprolol plasma concentration–time profiles belong to studies that stratified their subjects by CYP2D6 activity score or phenotype. These studies either provided the activity score for the investigated population (three studies), the CYP2D6 phenotype (two studies), or comprehensive information on the *CYP2D6* genotype of all individuals (10 studies). To simulate the latter studies, mean activity scores were calculated according to current recommendations [33]. The good performance of the final metoprolol DGI model is demonstrated in Figure 4, showing predicted metoprolol plasma concentration-time profiles of populations with different CYP2D6 activity scores, compared with their corresponding observed data. Plots documenting the model performance for all 15 metoprolol DGI profiles found in the literature are provided in Supplementary Section S3.2.

**Figure 5:**
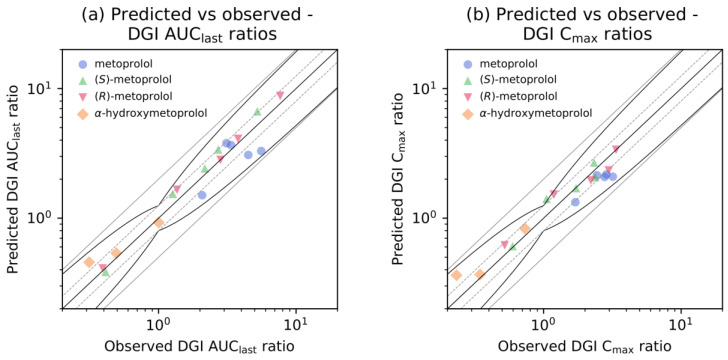
Predicted versus observed metoprolol DGI ratios. Comparison of predicted versus observed (**a**) DGI AUC_last_ ratios and (**b**) DGI C_max_ ratios for all analyzed metoprolol CYP2D6 DGI studies. The straight black line indicates the line of identity, curved black lines show prediction success limits proposed by Guest et al. including 1.25-fold variability [52]. Solid grey lines indicate two-fold deviation, dashed grey lines show 1.25-fold deviation. Detailed information on all clinical studies as well as the plotted values are listed in Tables S2.2.1 and S3.3.2 of the Supplementary Materials. AUC_last_: area under the plasma concentration-time curve from the time of the first concentration measurement to the time of the last concentration measurement, C_max_: maximum plasma concentration, DGI: drug-gene interaction, vs: versus.

Predicted DGI AUC_last_ and C_max_ ratios were in very good agreement with the observed DGI ratios, demonstrating that the impact of the different CYP2D6 activity scores on the pharmacokinetics of racemic metoprolol, (*R*)-, and (*S*)-metoprolol and the metabolite α-hydroxymetoprolol was well described by the model. Specifically, 18 out of 18 AUC_last_ and 17 out of 18 C_max_ ratios were within the prediction success limits suggested by Guest et al. adopted for DGI evaluations [52], as visualized in Figure 5. Predicted DGI AUC_last_ ratios show an overall GMFE of 1.21 (range 1.00–1.69), while predicted DGI C_max_ ratios showed an overall GMFE of 1.21 (range 1.00–1.56). The predicted and observed ratios and corresponding predicted to observed DGI AUC_last_ and C_max_ ratios for all studies are provided in Supplementary Table S3.3.2.

### 3.3. Metoprolol Dose Adaptation for CYP2D6 DGIs

**Figure 6:**
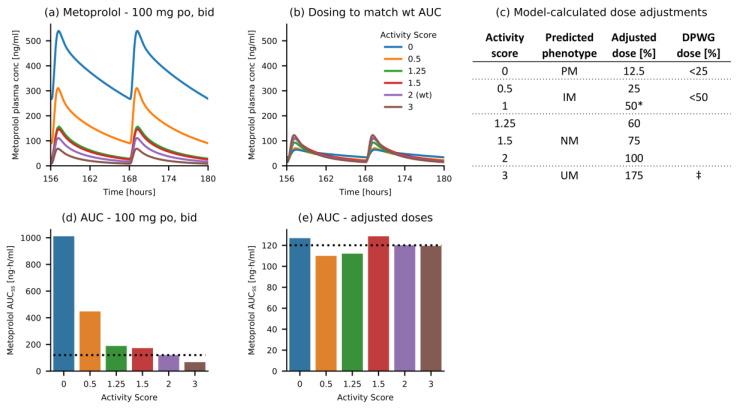
Model-based CYP2D6 DGI dose recommendations. (**a**) Simulations of metoprolol exposure in individuals with different CYP2D6 activity scores, all administered with 100 mg metoprolol twice daily. (**b**) Simulations of metoprolol exposure in individuals with different CYP2D6 activity scores, administered with the model-based dose recommendations. Doses were adjusted to match the AUC_168–180 h_ of 100 mg metoprolol twice daily in AS = 2 (wt) individuals. (**c**) Model-based dose adjustments, compared to the DPWG guideline recommendations for metoprolol [28]. (**d**) Metoprolol AUC_168–180 h_ values for administration of 100 mg twice daily to individuals with different CYP2D6 activity scores. (**e**) Metoprolol AUC_ss_ values for administration of the model-based dose recommendations to individuals with different CYP2D6 activity scores. The dotted horizontal line marks the wt AUC_ss_. *: value interpolated due to a lack of clinical studies with AS = 1, ‡: dose titration or change of medication recommended, AS: activity score, AUC_ss_: area under the plasma concentration-time curve during steady state (168–180 h), bid: twice daily, DPWG: Dutch Pharmacogenetics Working Group, IM: intermediate metabolizer, NM: normal metabolizer, PM: poor metabolizer, po: oral, UM: ultrarapid metabolizer, wt: wild type.

The developed metoprolol CYP2D6 DGI model was applied to calculate dose adaptations for individuals with different CYP2D6 activity scores. Simulated doses for “variant” activity scores were adapted in a stepwise approach until the AUC during steady-state (AUC_ss_) matched the AUC_ss_ (±10%) of a 100 mg twice daily metoprolol regimen in AS = 2 (wildtype) subjects. Predictions of plasma concentration-time profiles for individuals with different activity scores, all administered with 100 mg of metoprolol tartrate twice daily, are shown in Figure 6a. Simulations for different activity scores after dose adaptation are shown in Figure 6b. The resulting model-based dose adaptations compared to the Dutch Pharmacogenetics Working Group (DPWG) guideline recommendations for metoprolol [28] are shown in Figure 6c. The corresponding AUC_ss_ values before (Figure 6d) and after (Figure 6e) dose adaptation are visualized in the lower panel.

## 4. Discussion 

In this study, a whole-body PBPK model of metoprolol, including separate representations of its (*R*)- and (*S*)-enantiomers and the metabolite α-hydroxymetoprolol, was built and carefully evaluated to dynamically predict drug plasma concentrations over a wide dosing range (5–200 mg). Moreover, the model was extended to describe the impact of different CYP2D6 activity scores on the pharmacokinetics of racemic metoprolol, (*R*)-metoprolol, (*S*)-metoprolol, and α-hydroxymetoprolol.

Previously published metoprolol PBPK models were mostly developed for different applications. Indeed, two models investigated the effects of pregnancy [24,27] and one model analyzed the effects of investigational formulations [26]. A fourth published minimal PBPK-PD model of metoprolol was built to describe the impact of CYP2D6 DGIs on metoprolol plasma concentration profiles and heart rate. The DGI was implemented for three “traditional” phenotypes (poor, normal and ultrarapid metabolizers). This model, however, did not further differentiate the CYP2D6 activity between AS = 0 and AS = 2 [25]. Our model is the first to integrate current knowledge on CYP2D6 activity to accurately predict the impact of CYP2D6 DGIs over a wide range of activity scores. Moreover, this model is the first PBPK model of metoprolol to include metoprolol enantiomers (and enantiospecific CYP2D6 metabolism), as well as the active metabolite α-hydroxymetoprolol.

The limitations of the presented model are related to the incompleteness of published knowledge and data. Our model focused on CYP2D6 activity scores as opposed to CYP2D6 genotypes. Grouping genotypes by activity scores was necessary, due to the limited amount of data available on the enzyme kinetics of the >100 different CYP2D6 isoforms [53]. Consequently, the model is not able to further differentiate between different genotypes within the same activity score group (e.g., between *1/*1, *1/*2, and *2/*2, which all belong to the AS = 2 group) [7]. The primary aim of this model, namely the characterization, description, and prediction of metoprolol exposure in individuals with *CYP2D6* polymorphisms to enable model-informed precision dosing, was met [54]. As more data (in vitro and clinical) regarding the CYP2D6 activity of the different individual genotypes emerge, the model can be easily extended for an even finer graduation of the CYP2D6 activity, to differentiate between genotypes within the same activity score group. 

In addition, although the different CYP2D6 metabolic reactions (O-demethylation and α-hydroxylation of both (*R*)-metoprolol and (*S*)-metoprolol) were successfully implemented using K_m_ values from in vitro literature [39], these K_m_ values were assumed to be the same across all CYP2D6 activity scores. Using metoprolol as the substrate, only three genotype-specific in vitro K_m_ values (*1, *2 and *17 isoforms), could be obtained from literature (metoprolol α-hydroxylation and *O*-demethylation), showing a slightly higher K_m_ for the *17 allele (AS = 0.5) [8]. Other studies reported no clear trend of K_m_ values using a wide range of CYP2D6 substrates to investigate the enzyme kinetics of the reduced-function alleles *10 and *17 in comparison to the wildtype *1 allele [55]. Hence, due to an insufficient amount of data, the same K_m_ values were used in the model across all activity scores. The final optimized k_cat, rel_ values increased with increasing activity scores, reflecting an apparent correlation of metoprolol oral clearance with the CYP2D6 activity score [9]. Plasma concentration–time profiles and DGI AUC_last_ and C_max_ ratios of all analyzed clinical studies were well described by the final model. 

The enzymes CYP2B6, CYP2C9 and CYP3A4 have also been found to metabolize metoprolol in vitro [14]. However, the fractions metabolized by these CYP enzymes in vivo, or which of those enzymes is the second most relevant enzyme for metoprolol metabolism besides CYP2D6, is not known (clinical DDI studies with fluconazole, ketoconazole or other strong CYP3A4 inhibitors could not be found in the literature). In two of the previously published metoprolol PBPK models, a CYP3A4-dependent clearance process was implemented [24,25]. Yet, the formation of O-demethylmetoprolol and α-hydroxymetoprolol in human liver microsomes were less impacted by inhibition of CYP3A4 than by inhibition of CYP2C9 or CYP2B6 [14]. However, as CYP2D6 is estimated to account for >70% of metoprolol oral clearance [43], the impact of variations in CYP2B6, CYP2C9 or CYP3A4 enzymatic activity on metoprolol PK was considered negligible. Moreover, model input parameters such as CYP2B6, CYP2C9, or CYP3A4 K_m_ and k_cat_, that would be necessary for a mechanistic implementation of the respective metabolic pathways, are not available in the literature. Consequently, the authors decided to incorporate an unspecific hepatic clearance process in addition to the CYP2D6-dependent pathways. 

The final metoprolol PBPK model was applied to generate dose adaptations for populations with different CYP2D6 activity scores. While it is generally acknowledged that metoprolol exposure is mainly determined by the CYP2D6 activity score [56,57], there is no consensus in the literature on whether increased metoprolol plasma concentrations in poor and intermediate metabolizers result in a higher incidence of adverse drug reactions [58,59,60,61].

The model-based dose recommendations calculated for CYP2D6 DGIs were well in line with the recommendations provided by the DPWG [28], except for the poor metabolizers, where this analysis suggests even lower doses than the Dutch guidance document. Adapting a patients’ metoprolol dose based on the CYP2D6 activity score will decrease the occurrence of adverse drug reactions or therapy failure [56,59] and consequently help to provide more safe and efficient personalized dosing regimens. Future possible applications of the newly developed PBPK model include the prediction of CYP2D6 DDI effects on metoprolol pharmacokinetics or scaling of the metoprolol model to special populations such as pediatric patients, geriatric patients, or patients with renal or hepatic impairment. 

## 5. Conclusions

A whole-body parent-metabolite PBPK model of metoprolol and its enantiomers was developed to predict racemic metoprolol, (*R*)-metoprolol, (*S*)-metoprolol, and α-hydroxymetoprolol plasma concentration–time profiles. The model focused on CYP2D6 activity score-dependent metabolism and has been utilized to calculate dose adaptations in populations with various CYP2D6 activities and genotypes. The Supplementary Materials of this manuscript provide an in-depth documentation and evaluation of the final model and the PBPK model file will be made publicly available in the OSP repository. The model can be applied to generate dose adaption for patients with different CYP2D6 activity scores, to complement and refine the recommendations by existing guidelines and facilitate personalized medicine. Due to the mechanistic implementation of the human physiology and important pharmacokinetic pathways, the model allows for knowledge-based scaling to special populations and can serve as the basis for future investigations of CYP2D6 DDI scenarios. 

## Figures and Tables

**Table 1:** CYP2D6 activity score assignment according to [33].

Activity Score	Projected Phenotype	Examples of Relevant *CYP2D6* Genotypes
0	PM	*3/*3, *3/*4, *4/*4, *5/*6
0.25	IM	*4/*10, *5/*10
0.5		*4/*41, *5/*17, *10/*10
0.75		*17/*10, *41/*10
1		*1/*4, *2/*5, *17/*17, *17/*41
1.25	NM	*1/*10, *2/*10, *35/*10
1.5		*1/*41, *2/*17, *35/*41
2		*1/*1, *1/*2, *2/*35
2.25		*1x2/*17, *35x2/*41
>2.25	UM	*1/*1x3, *1/*35x2, *2x2/*9

CYP2D6: Cytochrome P450 2D6, IM: intermediate metabolizer, NM: normal metabolizer, PM: poor metabolizer, UM: ultrarapid metabolizer.

**Table 2:** (*R*)- and (*S*)-metoprolol drug-dependent model parameters.

Parameter	Unit	(*R*)-Metoprolol				(*S*)-Metoprolol				Description
		Value	Source	Literature	Reference	Value	Source	Literature	Reference	
MW	g/mol	267.36	Lit.	267.36	[34]	267.36	Lit.	267.36	[34]	Molecular weight
pK_a_ (base)	-	9.7	Lit.	9.70	[34]	9.7	Lit.	9.70	[34]	Acid dissociation constant
Solubility tart. (pH 7.4)	g/mL	1.00	Lit.	1.00	[35]	1.00	Lit.	1.00	[35]	Solubility
Solubility succ. (pH 5.5)	g/mL	0.16	Lit.	0.16	[36]	0.16	Lit.	0.16	[36]	Solubility
logP	-	1.77	Lit.	1.77	[37]	1.77	Lit.	1.77	[37]	Lipophilicity
f_u_	%	88	Lit.	88	[38]	88	Lit.	88	[38]	Fraction unbound
CYP2D6 K_m_ ⭢ αHM	µmol/L	10.08	Lit.	10.08 ^‡^	[39]	10.75	Lit.	10.75 ^‡^	[39]	Michaelis-Menten constant
CYP2D6 k_cat_ ⭢ αHM	1/min	6.02	Optim. ^†^	7.50	[39]	6.55	Optim. ^†^	8.27	[39]	Catalytic rate constant
CYP2D6 K_m_ ⭢ ODM	µmol/L	8.82	Lit.	8.82 ^‡^	[39]	12.43	Lit.	12.43 ^‡^	[39]	Michaelis-Menten constant
CYP2D6 k_cat_ ⭢ ODM	1/min	9.87	Optim. ^†^	12.30	[39]	8.21	Optim. ^†^	10.37	[39]	Catalytic rate constant
CL_hep., unsp._	1/min	0.08	Optim.	-	-	0.09	Optim.	-	-	Unspecific hepatic clearance
GFR fraction	-	1.00	Asm.	-	-	1.00	Asm.	-	-	Filtered drug in the urine
EHC continuous fraction	-	1.00	Asm.	-	-	1.00	Asm.	-	-	Bile fraction cont. released
Intestinal permeability	cm/min	4.14 × 10^−5^	Optim.	1.12 × 10^−5^	Calc. [40]	4.14 × 10^−5^	Optim.	1.12 × 10^−5^	Calc. [40]	Transcellular intestinal perm.
Cellular permeability	cm/min	4.64 × 10^−3^	Calc.	PK-Sim	[32]	4.64 × 10^−3^	Calc.	PK-Sim	[32]	Perm. into the cellular space
Partition coefficients	-	Diverse	Calc.	R&R	[41,42]	Diverse	Calc.	R&R	[41,42]	Cell to plasma partitioning
NR Weibull time parameter	min	12.31	Optim.	-	[43,44]	12.31	Optim.	-	[43,44]	Dissolution time (50%)
NR Weibull shape parameter	-	0.72	Optim.	-	[43,44]	0.72	Optim.	-	[43,44]	Dissolution profile shape
CR Weibull time parameter	min	331.92	Optim.	-	[45]	331.92	Optim.	-	[45]	Dissolution time (50%)
CR Weibull shape parameter	-	1.53	Optim.	-	[45]	1.53	Optim.	-	[45]	Dissolution profile shape

-: not available, ^†^: CYP2D6 k_cat_ values were optimized in a fixed ratio (k_cat_ **⭢** αHM:k_cat_ **⭢** ODM) equivalent to the ratio of reported k_cat_ values [39], ^‡^: in vitro values corrected for binding in the assay, using estimated fraction unbound to microsomal protein (f_u, mic, estimated_ = 84%) [46], αHM: α-hydroxymetoprolol, asm.: assumed, calc.: calculated, cont.: continuously, CR: controlled release, CYP2D6: cytochrome P450 2D6, EHC: enterohepatic circulation, GFR: glomerular filtration rate, hep.: hepatic, lit.: literature, NR: normal release, ODM: *O*-demethylmetoprolol, optim.: optimized, perm. permeability, PK-Sim: PK-Sim standard calculation method, R&R: Rodgers and Rowland calculation method, succ.: metoprolol succinate, tart.: metoprolol tartrate, unsp.: unspecific.

**Table 3:** Optimized k_cat, rel_ values for the different modeled CYP2D6 activity scores.

Activity Score	(*R*)-Metoprolol		(*S*)-Metoprolol		k_cat, rel_
	k_cat_ ⭢ αHM	k_cat_ ⭢ ODM	k_cat_ ⭢ αHM	k_cat_ ⭢ ODM	
0	0.00 1/min	0.00 1/min	0.00 1/min	0.00 1/min	0%
0.5	1.65 1/min	2.70 1/min	1.82 1/min	2.27 1/min	19%
1.25	5.73 1/min	9.40 1/min	6.30 1/min	7.89 1/min	64%
1.5	6.38 1/min	10.48 1/min	7.03 1/min	8.81 1/min	72%
2	10.17 1/min	16.69 1/min	11.19 1/min	14.02 1/min	100%
3	19.03 1/min	31.22 1/min	20.93 1/min	26.23 1/min	213%

αHM: α-hydroxymetoprolol, k_cat_: catalytic rate constant, k_cat, rel_: catalytic rate constant relative to activity score = 2, ODM: *O*-demethylmetoprolol.

## Supplementary Materials

The following are available online at https://www.mdpi.com/1999-4923/12/12/1200/s1, Table S2.2.1: Metoprolol study table, Table S2.3.2: (*R*)-and(*S*)-metoprolol drug-dependent parameters, Table S2.4.3: α-hydroxymetoprolol drug-dependent parameters, Figure S2.5.1: Metoprolol plasma concentrations. Model predictions of metoprolol and its metabolite α-hydroxymetoprolol plasma concentration-time profiles of intravenous studies of the training and test datasets, compared to observed data (semilogarithmic representation), Figure S2.5.2: Metoprolol plasma concentrations. Model predictions of metoprolol and its metabolite α-hydroxymetoprolol plasma concentration-time profiles of oral studies of the training and test datasets, compared to observed data (semilogarithmic representation), Figure S2.5.3: Metoprolol plasma concentrations, Figure S2.5.4: Metoprolol enantiomers plasma concentrations. Model predictions of (*R*)-metoprolol and (*S*)-metoprolol plasma concentration-time profiles of oral studies of the trainingand test datasets, compared to observed data (semilogarithmic representation), Figure S2.5.5: Metoprolol plasma concentrations, Figure S2.5.6: Metoprolol plasma concentrations, Figure S2.5.7: Metoprolol plasma concentrations, Figure S2.5.8: Metoprolol enantiomers plasma concentrations, Figure S2.6.9: Plasma concentrations goodness-of-fit plots of the final metoprolol model, Figure S2.6.10: Plasmaconcentrationsgoodness-of-fitplotsofthefinalmetoprololmodel, Table S2.6.4: Mean relative deviation of plasma concentration predictions (metoprolol, αhydroxymetoprolol), Table S2.6.5: Mean relative deviation of plasma concentration predictions ((*R*)-metoprolol, (*S*)-metoprolol), Figure S2.6.11: AUC_last_ values goodness-of-fit plots for the final metoprolol model, Figure S2.6.12: AUC_last_ goodness-of-fit plots for the final metoprolol model, Figure S2.6.13: C_max_ values goodness-of-fit plots for the final metoprolol model, Figure S2.6.14: AUC_last_ goodness-of-fit plots for the final metoprolol model, Table S2.6.6: Predicted and observed AUC_last_ and C_max_ values (metoprolol, α-hydroxymetoprolol), Table S2.6.7: Predictedandobserved AUC_last_ and C_max_ values ((*R*)-metoprolol,(*S*)-metoprolol), Figure S2.6.15: Sensitivity analysis of the (*R*)-metoprolol (upper panel) and (*S*)-metoprolol (lower panel) model, Table S3.1.1: k_cat_, _rel_ values for the different CYP2D6 activity scores, Figure S3.2.1: Metoprolol plasma concentrations of the modeled CYP2D6 drug-gene interaction, Figure S3.2.2: Metoprolol plasma concentrations of the modeled CYP2D6 drug-gene interaction, Figure S3.2.3: Metoprolol plasma concentrations of the modeled CYP2D6 drug-gene interaction, Figure S3.2.4: Metoprolol plasma concentrations of the modeled CYP2D6 drug-gene interaction, Figure S3.2.5: Metoprolol plasma concentrations of the modeled CYP2D6 drug-gene interaction, Figure S3.2.6: Metoprolol plasma concentrations of the modeled CYP2D6 drug-gene interaction., Figure S3.2.7: Metoprolol plasma concentrations of the modeled CYP2D6 drug-gene interaction, Figure S3.2.8: Metoprolol plasma concentrations of the modeled CYP2D6 drug-gene interaction, Figure S3.2.9: Metoprolol plasma concentrations of the modeled CYP2D6 drug-gene interaction, Figure S3.2.10: Metoprolol plasma concentrations of the modeled CYP2D6 drug-gene interaction, Figure S3.2.11: Metoprolol plasma concentrations of the modeled CYP2D6 drug-gene interaction, Figure S3.2.12: Metoprolol plasma concentrations of the modeled CYP2D6 drug-gene interaction, Figure S3.3.13: Predicted versus observed metoprolol DGI ratios. Comparison of predicted versus observed AUC_last_ ratios (a) and C_max_ ratios (b) for metoprolol CYP2D6 DGI-studies, Table S3.3.2: Geometric mean fold error of predicted metoprolol DGI AUC_last_ andC_max_ ratios, Table S4.0.1: System-dependent parameters.
