# Supplementary material for: Physiologically Based Pharmacokinetic Modeling of Metoprolol Enantiomers and α-Hydroxymetoprolol to Describe CYP2D6 Drug-Gene Interactions
Source: Pharmaceutics. 2020 Dec 11;12(12):1200. doi: 10.3390/pharmaceutics12121200 (PMC7763912; doi:10.3390/pharmaceutics12121200)
Supplement: Supplementary file 1 [file pharmaceutics-12-01200-s001.pdf]

pharmaceutics

MDPI

Supplementary Materials

Physiologically Based Pharmacokinetic Modeling of Metoprolol Enantiomers and α-Hydroxymetoprolol to Describe CYP2D6 Drug-Gene Interactions

Simeon Rüdesheim[1,2], Jan-Georg Wojtyniak[1,2], Dominik Selzer[1], Nina Hanke[1], Felix Mahfoud[3,4], Matthias Schwab[2,5,6], Thorsten Lehr[1], *

1 Clinical Pharmacy, Saarland University, 66123 Saarbrücken, Germany; simeon.ruedesheim@uni-saarland.de (S.R.); jangeorg.wojtyniak@uni-saarland.de (J.-G.W.); dominik.selzer@uni-saarland.de (D.S.); n.hanke@mx.uni-saarland.de (N.H.)
2 Dr. Margarete Fischer-Bosch-Institute of Clinical Pharmacology, 70376 Stuttgart, Germany; matthias.schwab@ikp-stuttgart.de
3 Department of Internal Medicine III, Cardiology, Angiology, Intensive Care Medicine, Saarland University Medical Center and Saarland University Faculty of Medicine, 66421 Homburg/Saar, Germany; felix.mahfoud@uks.eu
4 Institute for Medical Engineering and Science, Massachusetts Institute of Technology, 02139 Cambridge, MA, USA
5 Departments of Clinical Pharmacology, Pharmacy and Biochemistry, University Tübingen, 72076 Tübingen, Germany
6 Cluster of Excellence iFIT (EXC2180) "Image-Guided and Functionally Instructed Tumor Therapies", University of Tübingen, 72076 Tübingen, Germany
* Correspondence: thorsten.lehr@mx.uni-saarland.de; Tel.: +49-681-302-70255

Received: 10 November 2020; Accepted: 5 December 2020; Published: 11 December 2020

Funding: F.M. is supported by Deutsche Gesellschaft für Kardiologie (DGK), and Deutsche Forschungsgemeinschaft (SFB TRR219). M.S. was supported by by the Robert Bosch Stiftung (Stuttgart, Germany), the European Commission Horizon 2020 UPGx grant 668353, a grant from the German Federal Ministry of Education and Research (BMBF 031L0188D), and the Deutsche Forschungsgemeinschaft (DFG, German Research Foundation) under Germany's Excellence Strategy-EXC 2180—390900677. T.L. was supported by the German Federal Ministry of Education and Research (BMBF, Horizon 2020 INSPIRATION grant 643271), under the frame of ERACoSysMed The APC was funded by the German Research Foundation (DFG) and Saarland University within the funding program "Open Access Publishing".

Conflict of Interest: J.-G.W. is an employee of Boehringer Ingelheim Pharma GmbH & Co. KG. F.M. received scientific support and speaker honoraria from Bayer, Boehringer Ingelheim, Medtronic and ReCor Medical. S.R., N.H., D.S., M.S. and T.L. declare that they have no conflict of interest. The funders had no role in the design of the study; in the collection, analyses, or interpretation of data; in the writing of the manuscript, or in the decision to publish the results.

Contents

S1 Physiologically based pharmacokinetic (PBPK) modeling 4
S1.1 PBPK model building 4
S1.1.1 PBPK model building 4
S1.1.2 Metoprolol formulations 5
S1.1.3 Virtual individuals 5
S1.1.4 Virtual populations 5
S1.2 PBPK model evaluation 6
S1.2.1 PBPK model evaluation 6
S1.2.2 PBPK model sensitivity analysis 6
S1.3 CYP2D6 DGI Modeling 7
S1.3.1 Implementation of CYP2D6 DGI 7
S1.3.2 DGI Model Evaluation 7

S2 PBPK modeling of metoprolol 9
S2.1 Metoprolol model development 9
S2.2 Clinical studies 11
S2.3 Drug-dependent parameters: (*R*)- and (*S*)-metoprolol 13
S2.4 Drug-dependent parameters: α-hydroxymetoprolol 14
S2.5 Plasma profiles 15
S2.5.1 Semilogarithmic plots 15
S2.5.2 Linear plots 19
S2.6 Model evaluation 23
S2.6.1 Plasma concentrations goodness-of-fit plots 23
S2.6.2 Mean relative deviation of plasma concentration predictions (metoprolol, α-hydroxymetoprolol) 25
S2.6.3 Mean relative deviation of plasma concentration predictions ((*R*)-metoprolol, (*S*)-metoprolol) 27
S2.6.4 AUC_{last} and C_{max} values goodness-of-fit plots 28
S2.6.5 Geometric mean fold error of predicted AUC_{last} and C_{max} values (metoprolol, α-hydroxymetoprolol) 31
S2.6.6 Geometric mean fold error of predicted AUC_{last} and C_{max} values ((*R*)-metoprolol, (*S*)-metoprolol) 33
S2.6.7 Sensitivity analysis 34

S3 Metoprolol CYP2D6 DGI model 36
S3.1 Metoprolol k_{cat} values for the modeled activity scores 36
S3.2 Plasma profiles 37
S3.2.1 Semilogarithmic plots 37
S3.2.2 Linear plots 40
S3.3 Model evaluation 43
S3.3.1 Metoprolol CYP2D6 DGI AUC_{last} and C_{max} ratio plots 43

S3.3.2 Geometric mean fold error of predicted metoprolol DGI AUC_{last} and C_{max} ratios 44

S4 System-dependent parameters **45**

S5 Abbreviations **46**

S1 Physiologically based pharmacokinetic (PBPK) modeling

S1.1 PBPK model building

S1.1.1 PBPK model building

Physiologically based pharmacokinetic (PBPK) modeling and model parameter optimization (Monte Carlo algorithm) were performed using PK-Sim® and MoBi® (Open Systems Pharmacology Suite 9.1). Published clinical study data were digitized with GetData Graph Digitizer 2.26.0.20 (©S. Fedorov) according to best practices [56]. For calculation of pharmacokinetic parameters and model performance metrics as well as generation of figures Python (version 3.7.4, Python Software Foundation, Wilmington, DE, USA) and Visual Studio Code (version 1.49.1, Microsoft Corporation, Redmond, WA, USA) were used. PBPK model building was initiated with an extensive literature search to gather information on metoprolol absorption, distribution, metabolism and excretion (ADME) processes, to obtain physicochemical data and to collect clinical studies of intravenous and oral administration of metoprolol, in single- and multiple-dose regimens, performed in healthy individuals. Subsequently, plasma concentration-time profiles from the published clinical studies were digitized and split into a training dataset, for model building and a test dataset, for model evaluation (see Table S2.2.1 for information on all studies). Studies for model training were selected to include different routes of administration (intravenous and oral), a wide range of administered doses, single- and multiple-dose regimens as well as stratification for *cytochrome P450 2D6 (CYP2D6)* genotype or activity score. The training dataset was used for estimation of model input parameters which could not be obtained from literature. The final model parameters for metoprolol enantiomers and α-hydroxymetoprolol are provided in Tables S2.3.2 and S2.4.3, respectively. The metoprolol enantiomer PBPK model was built in a stepwise approach; first, appropriate quantitative structure-activity relationship (QSAR) methods to estimate the cellular permeabilities and partition coefficients were selected by minimizing the residual sum of squares of simulations of intravenous metoprolol administration and their observed data. Subsequently, studies of orally administered metoprolol in poor metabolizers (PMs) were used to optimize parameters independent of CYP2D6 metabolism. Finally, *(R)*- and *(S)*-enantiomer CYP2D6 catalytic rate constant (k_{cat}) values were optimized for studies of the training dataset where the volunteers were either normal metabolizers (NMs) or not phenotyped.

S1.1.2 Metoprolol formulations

The weibull function was implemented according to Equations S1 and S2 [28] to describe the dissolution process for different solid metoprolol formulations.

$$m = 1 - \exp\left(\frac{-(t - T_{lag})^{\beta}}{\alpha}\right) \tag{S1}$$

$$\alpha = (T_d)^{\beta} \tag{S2}$$

where m = fraction of dissolved drug at time t, T_{lag} = lag time before the onset of dissolution, α = scale parameter, β = shape parameter, T_d = time needed to dissolve 63% of the formulation.

The final Weibull shape parameters and Weibull time parameters (50% dissolved) for all solid formulations used in the metoprolol PBPK-model are given in Table S2.3.2.

S1.1.3 Virtual individuals

The PBPK model was built based on data from healthy individuals, using the reported sex, ethnicity and mean values for age, weight and height from each study protocol. If no demographic information was provided, the following default values were substituted: male, European, 30 years of age, 73 kg body weight and 176 cm body height (characteristics from the PK-Sim® population database ([35, 49, 52]). CYP2D6 was implemented in accordance with literature, using the PK-Sim® expression database to define their relative expression in the different organs of the body [38]. Details on the implementation of CYP2D6 are summarized in Section S4.

S1.1.4 Virtual populations

For population simulations, virtual populations of 100 individuals were created based on the population characteristics stated in the respective publication. If no information was provided in the publication, populations based on european male individuals aged 20–50 years were assumed. Metrics were generated (depending on ethnicity) from one of the following databases; American: Third National Health and Nutrition Examination Survey (NHANES) [35] database, Asian: Tanaka model [49], European: International Commission on Radiological Protection (ICRP) database [52]. In the generated virtual populations, system-dependent parameters such as weight, height, organ volumes, blood flow rates, tissue compositions, etc. were varied by the implemented algorithm in PK-Sim® within the limits of the databases listed above [35, 49, 52]. Since study populations were grouped by their CYP2D6 activity score or phenotype, no variability in CYP2D6 reference concentrations was assumed for population simulations. Reference concentrations of implemented proteins as well as their relative expression are provided in Section S4.

S1.2 PBPK model evaluation

S1.2.1 PBPK model evaluation

Model evaluation was carried out with different methods based on the clinical data of the test dataset. The population predicted plasma concentration-time profiles were compared to the data observed in the clinical studies. Furthermore, predicted plasma concentration values of all studies were compared to the observed plasma concentrations in goodness-of-fit plots. In addition, the model performance was evaluated by comparison of predicted to observed area under the plasma concentration-time curve (AUC) from the time of the first concentration measurement to the last time point of concentration measurement (AUC_{last}) and peak plasma concentration (C_{max}) values. As quantitative performance measures, a mean relative deviation (MRD) of the predicted plasma concentrations for all observed and the corresponding predicted plasma concentrations as well as geometric mean fold errors (GMFEs) of the AUC_{last} and C_{max} values were calculated according to Equation S3 and Equation S4, respectively.

$$\mathrm{MRD} = 10^x;\, x = \sqrt{\frac{\sum_{i=1}^{k}(\log_{10}\hat{c}_i - \log_{10}c_i)^2}{k}} \tag{S3}$$

where $\hat{c}_i$ = ith predicted plasma concentration, c_i = ith observed plasma concentration and k = number of observed values.

$$\mathrm{GMFE} = 10^x;\, x = \frac{\sum_{i=1}^{m}\left|\log_{10}\left(\frac{\hat{\rho}_i}{\rho_i}\right)\right|}{m} \tag{S4}$$

where $\hat{\rho}_i$ = ith predicted plasma AUC_{last} or C_{max} value, ρ_i = ith observed plasma AUC_{last} or C_{max} value and m = number of studies.

S1.2.2 PBPK model sensitivity analysis

Sensitivity of the final models to single parameter changes (local sensitivity analysis) was calculated as relative change of the $AUC_{0–24}$. Sensitivity analysis was carried out using a relative perturbation of 1000% (variation range 10.0, maximum number of 9 steps). Parameters were included into the analysis if they have been optimized, if they are associated with optimized parameters or if they might have a strong impact due to calculation methods used in the model. Sensitivity to a parameter was calculated as the ratio of the relative change of the simulated $AUC_{0–24\,h}$ to the relative variation of the parameter according to Equation S5:

$$S = \frac{\Delta AUC_{0-24\,h}}{\Delta p} \times \frac{p}{AUC_{0-24\,h}} \tag{S5}$$

where S = sensitivity of the AUC to the examined model parameter, $\Delta AUC_{0-24\,h}$ = change of the $AUC_{0–24\,h}$, $AUC_{0-24\,h}$ = simulated $AUC_{0–24\,h}$ with the original parameter value, Δp = change of the examined parameter value, p = original parameter value.

A sensitivity of +1.0 signifies that a 10% increase of the examined parameter value causes a 10% increase of the simulated $AUC_{0-24\,h}$. The results of the sensitivity analysis are provided in Section S2.6.7

S1.3 CYP2D6 DGI Modeling

S1.3.1 Implementation of CYP2D6 DGI

The model training dataset included 11 plasma concentration-time profiles from studies that reported the CYP2D6 activity scores of their study subjects, ranging from 0 (PM) to 3 (ultrarapid metabolizer (UM)). These studies were utilized to optimize catalytic rate constant relative to CYP2D6 activity score (AS)=2 ($k_{cat,\,rel}$) values for the different CYP2D6 activity scores. CYP2D6 poor metabolizers (AS=0) were assumed to show no CYP2D6 activity (0%), whereas populations with two wildtype alleles (AS=2) were used as reference (100%) to calculate relative k_{cat} values according to Equation S6:

$$k_{cat,\ rel,\ AS=i} = \frac{k_{cat,\ AS=i}}{k_{cat,\ AS=2}} \cdot 100\% \tag{S6}$$

where $k_{cat,\ rel}$ = k_{cat} relative to AS=2 for the investigated AS, $k_{cat,\ AS=i}$ = k_{cat} for the investigated AS and $k_{cat,\ AS=2}$ = k_{cat} for AS = 2.

The identified values for both CYP2D6 pathways and both metoprolol enantiomers are listed in Table S3.1.1. CYP2D6 Michaelis-Menten constant (K_m) values were kept constant over the whole range of modeled activity scores. Since study populations were grouped by their CYP2D6 activity score or phenotype, no variability in CYP2D6 reference concentrations was implemented for population simulations (see Section S4 for details on the implementation of CYP2D6).

S1.3.2 DGI Model Evaluation

The drug-gene interaction (DGI) modeling performance was assessed by comparison of predicted versus observed plasma concentration-time profiles of racemic metoprolol, its enantiomers and α-hydroxymetoprolol (see Chapter S3). Furthermore, predicted DGI AUC_{last} ratios (Equation S7) and DGI C_{max} ratios (Equation S8) were evaluated.

$$DGI\ AUC_{last}\ ratio = \frac{AUC_{last,\ DGI}}{AUC_{last,\ reference}} \tag{S7}$$

where $AUC_{last,\ DGI}$ = AUC_{last} of variant activity score or phenotype, $AUC_{last,\ reference}$ = AUC_{last} of AS=2 or normal metabolizer phenotype.

$$DGI\ C_{max}\ ratio = \frac{C_{max,\ DGI}}{C_{max,\ reference}} \tag{S8}$$

where $C_{max,\ DGI}$ = C_{max} of variant activity score or phenotype, $C_{max,\ reference}$ = C_{max} of AS=2 or normal metabolizer phenotype. As a quantitative measure of the prediction accuracy, GMFE values of the predicted DGI AUC_{last} ratios and DGI C_{max} ratios were calculated according to Equation S4 and are given in Table S3.3.2.

S2 PBPK modeling of metoprolol

S2.1 Metoprolol model development

Metoprolol is the most frequently administered beta-blocker in the U.S. with well over 50 million total prescriptions per year [10]. It is used in the treatment of hypertension, angina pectoris, heart failure, arterial fibrillation as well as acute myocardial infarction [36]. Metoprolol is listed by the U. S. Food and Drug Administration (FDA) as a moderately sensitive substrate for clinical drug-drug interaction (DDI) studies as it is predominantly metabolized by CYP2D6 [51]. Metoprolol is a Biopharmaceutics Classification System (BCS) Class I drug, characterized by high permeability and high solubility. After its rapid absorption, metoprolol undergoes extensive first-pass metabolism, reducing its bioavailability (BA) to 40% in CYP2D6 NMs, whereas BA approaches 100% in PMs [25]. Only 12% of metoprolol are bound to plasma proteins, primarily albumin [40]. *O*-demethylation, α-hydroxylation and *N*-dealkylation by CYP2D6 and, to lesser extents CYP2B6, CYP2C9, CYP3A4 are described as the pathways of metoprolol metabolism [5, 42]. Of the major metabolites, α-hydroxymetoprolol is of particular clinical interest, as it is pharmacologically active, exhibiting 10% of the β_1-blocking activity of metoprolol [8], and it is almost exclusively formed via CYP2D6 [29]. Therefore, α-hydroxymetoprolol/metoprolol urinary metabolic ratios are employed for CYP2D6 phenotyping [7]. Overall, CYP2D6 is estimated to be responsible for 80% of metoprolol metabolism in normal metabolizers [5]. Depending on the CYP2D6 phenotype, only 1.5–12% of orally administered metoprolol are excreted unchanged in urine [46]. Metoprolol is a chiral molecule, marketed as racemic mixture of (*R*)- and (*S*)-metoprolol, even though its enantiomers differ in their pharmacodynamic and pharmacokinetic properties. The (*S*)-enantiomer has been shown to be 33-fold more potent in blocking β_1-adrenoceptors in rats than the (*R*)-enantiomer [34]. Moreover, in UMs and NMs but not in PMs, the (*S*)-metoprolol AUC is significantly higher than the AUC of (*R*)-metoprolol, showing the enantiopreference of CYP2D6 towards the (*R*)-enantiomer [46].

A total of 48 clinical studies of intravenous or oral administration of metoprolol were used in the model development process, with doses ranging from 5–200 mg metoprolol in single or multiple dose regimens. Of the 48 studies, nine included measurements of the metabolite α-hydroxymetoprolol and 16 studies included measurements of the metoprolol enantiomers. Details on all studies used for PBPK modeling are given in Table S2.2.1. The four α-hydroxymetoprolol diastereomers were modeled as one single compound, due to a lack of enantiomeric differentiation in the published clinical data. For both metoprolol enantiomers, enantioselective metabolism via CYP2D6, an unspecific hepatic clearance (CL) process as well as passive glomerular filtration were implemented. Each of the metoprolol enantiomers can be metabolized via CYP2D6 to either produce α-hydroxymetoprolol or to generate other metabolites such as *O*-

demethylmetoprolol which were not included as separately modeled compounds. The metabolite α-hydroxymetoprolol is eliminated via an unspecific hepatic CL process. The drug-dependent model input parameters of the metoprolol enantiomers are presented in Table S2.3.2; the drug-dependent parameters of the α-hydroxymetoprolol model are given in S2.4.3.

The performance of the metoprolol model is demonstrated in semilogarithmic (Section S2.5.1) and linear plots (Section S2.5.2) of population simulations compared to observed plasma concentration-time profiles of all clinical studies. Furthermore, goodness-of-fit plots comparing all predicted to their corresponding observed plasma concentrations of metoprolol enantiomers, racemic metoprolol and α-hydroxymetoprolol (Figures S2.6.9 and S2.6.10) as well as MRD values for each study (see Tables S2.6.4 and S2.6.5) are presented. Moreover, correlation plots of predicted versus observed AUC_{last} (Figures S2.6.11 and S2.6.12) and C_{max} (Figures S2.6.13 and S2.6.14) values are shown, including calculated model GMFE values (Tables S2.6.6 and S2.6.7). Finally, a sensitivity analysis of a simulation of a single oral dose of 100 mg metoprolol tartrate, administered as a tablet in the fasted state was performed. The results of the sensitivity analysis are given in Section S2.6.7.

S2.2 Clinical studies

Table S2.2.1: Metoprolol study table

Route	Dose [mg]	n	Females [%]	Age [years]	Weight [kg]	Metabolite measured	Enantiomers measured	CYP2D6 activity	Dataset	Reference
iv (inf, 150 min, sd)	88.7	6	17	(23–29)	-	yes	no	-	test	Godbillon et al. 1985 [12]
iv (inf, 10 min, sd)	50	12	0	(19–26)	(60–98)	no	no	-	training	Kelly et al. 1985 [23]
iv (inf, 10 min, sd)	20	5	0	(23–28)	(62–70)	no	no	-	test	Johnsson et al. 1975 [22]
iv (inf, 10 min, sd)	15	5	0	(23–28)	(62–70)	no	no	-	test	Johnsson et al. 1975 [22]
iv (inf, 10 min, sd)	10	5	0	(23–28)	(62–70)	no	no	-	test	Johnsson et al. 1975 [22]
iv (inf, 5 min, sd)	10	6	0	(23–28)	-	no	no	-	test	Regårdh et al. 1980 [42]
iv (inf, 10 min, sd)	5	5	0	(23–28)	(62–70)	no	no	-	training	Regårdh et al. 1974 [41]
iv (inf, 10 min, sd)	5	5	0	(23–28)	(62–70)	no	no	-	test	Johnsson et al. 1975 [22]
po (tab, CR, daily)	200	15	27	(21–45)	-	no	no	-	training	Damy et al. 2004 [11]
po (tab, sd)	200	10	0	29 (24–40)	85	no	yes	p-NM	training	Johnson et al. 1996 a [20]
po (tab, sd)	200	10	0	29 (24–36)	82	no	yes	p-NM	training	Johnson et al. 1996 b [20]
po (tab, CR, sd)	200	15	27	(21–45)	-	no	yes	AS=1.5*	test	Parker et al. 2011 [39]
po (tab, sd)	100	4	0	-	-	yes	no	AS=2.0*	test	Bae et al. 2014 [3]
po (tab, sd)	100	3	0	-	-	yes	no	AS=0.5*	test	Bae et al. 2014 [3]
po (-, sd)	100	12	0	28 (21–35)	71 (62–82)	no	no	-	test	Bennett et al. 1982 [4]
po (tab, sd)	100	12	50	(22–34)	-	no	no	-	test	Chellingsworth et al. 1988 [9]
po (tab, bid)	100	12	0	(23–32)	-	no	no	-	test	Chellingsworth et al. 1988 [9]
po (tab, sd)	100	10	0	26 (20–36)	73 (59–96)	no	no	p-NM	test	Hamelin et al. 2000 [14]
po (tab, sd)	100	6	0	26 (20–36)	73 (59–96)	no	no	p-PM	test	Hamelin et al. 2000 [14]
po (tab, sd)	100	8	0	(20–29)	-	no	yes	g-NM	test	Hemeryck et al. 2000 [15]
po (tab, sd)	100	7	43	52 (29–68)	-	no	no	-	test	Houtzagers et al. 1982 [16]
po (tab, sd)	100	15	0	(19–23)	-	no	no	-	test	Jack et al. 1982 [18]

*: AS calculated from genotype provided in publication, AS: CYP2D6 activity score, bid: twice daily, CR: controlled release, g-: genotyped, inf: infusion, iv: intravenous, NM: normal metabolizer, p-: phenotyped, PM: poor metabolizer, po: oral, sd: single dose, sol: oral solution, tab: tablet
Values are given as arithmetic means, the range of values are given in parentheses

Table S2.2.1: Metoprolol study table *(continued)*

Route	Dose [mg]	n	Females [%]	Age [years]	Weight [kg]	Metabolite measured	Enantiomers measured	CYP2D6 activity	Dataset	Reference
po (tab, sd)	100	5	0	(23–28)	(62–70)	no	no	-	test	Johnsson et al. 1975 [22]
po (tab, sd)	100	16	0	25	65	no	yes	AS=2*	training	Huang et al. 1999 [17]
po (tab, sd)	100	12	0	24	65	no	yes	AS=1.25*	training	Huang et al. 1999 [17]
po (tab, sd)	100	12	0	24	63	no	yes	AS=0.5*	training	Huang et al. 1999 [17]
po (tab, sd)	100	6	22	23	67	yes	no	AS=2*	training	Jin et al. 2008 [19]
po (tab, sd)	100	7	22	23	67	yes	no	AS=1.25*	training	Jin et al. 2008 [19]
po (tab, sd)	100	15	22	23	67	yes	no	AS=0.5*	training	Jin et al. 2008 [19]
po (-, sd)	100	12	0	(19–26)	(60–98)	no	no	-	training	Kelly et al. 1985 [23]
po (-, bid)	100	12	0	(19–26)	(60–98)	no	no	-	training	Kelly et al. 1985 [23]
po (tab, sd)	100	18	0	29 (18–39)	79 (62–100)	yes	no	g-NM	test	Krösser et al. 2006 [27]
po (-, sd)	100	12	0	33 (19–55)	-	yes	no	g-NM	test	Krauwinkel et al. 2013 [26]
po (tab, bid)	100	10	0	26 (20–36)	84 (66–97)	no	yes	p-NM	test	Luzier et al. 1999 a [30]
po (tab, bid)	100	10	100	25 (21–35)	62 (54–77)	no	yes	p-NM	test	Luzier et al. 1999 b [30]
po (tab, bid)	100	15	27	(21–45)	-	no	yes	AS=1.5*	test	Parker et al. 2011 [39]
po (tab, CR, sd)	100	15	27	(21–45)	-	no	yes	AS=1.5*	test	Parker et al. 2011 [39]
po (tab, sd)	100	12	8	28 (25–37)	76 (70–80)	no	yes	AS=3	training	Seeringer et al. 2008 [25,46]
po (tab, sd)	100	13	0	28 (23–34)	77 (69–81)	no	yes	AS=2	training	Seeringer et al. 2008 [25,46]
po (tab, sd)	100	4	0	38 (29–40)	90 (77–101)	no	yes	AS=0	training	Seeringer et al. 2008 [25,46]
po (tab, sd)	100	16	100	27 (18–40)	60 (49–100)	no	yes	AS=1.5*	training	Sharma et al. 2005 [47]
po (-, sd)	100	4	100	27 (18–40)	60 (49–100)	no	yes	AS=0	training	Sharma et al. 2005 [47]
po (tab, sd)	50	5	0	(23–28)	(62–70)	no	no	-	test	Johnsson et al. 1975 [22]
po (-, sd)	50	10	0	28 (18–45)	82 (63–94)	no	no	-	test	Stout et al. 2011 [48]
po (tab, CR, sd)	50	10	0	28 (18–45)	82 (63–94)	no	no	-	test	Stout et al. 2011 [48]
po (tab, sd)	50	12	0	31	78	yes	no	AS=1.5*	test	Werner et al. 2003 [54]
po (tab, sd)	20	5	0	(23–28)	(62–70)	no	no	-	test	Johnsson et al. 1975 [22]
po (sol, sd)	5	5	0	(23–28)	(62–70)	no	no	-	training	Regårdh et al. 1974 [41]

*: AS calculated from genotype provided in publication, AS: CYP2D6 activity score, bid: twice daily, CR: controlled release, g-: genotyped, inf: infusion, iv: intravenous, NM: normal metabolizer, p-: phenotyped, PM: poor metabolizer, po: oral, sd: single dose, sol: oral solution, tab: tablet
Values are given as arithmetic means, the range of values are given in parentheses

S2.3 Drug-dependent parameters: (*R*)- and (*S*)-metoprolol

Table S2.3.2: (*R*)- and (*S*)-metoprolol drug-dependent parameters

		**(*R*)-Metoprolol**				**(*S*)-Metoprolol**				
Parameter	Unit	Value	Source	Literature	Reference	Value	Source	Literature	Reference	Description
MW	g/mol	267.36	Lit.	267.36	[24]	267.36	Lit.	267.36	[24]	Molecular weight
pKa (base)	-	9.70	Lit.	9.70	[24]	9.70	Lit.	9.70	[24]	Acid dissociation constant
Solubility tart. (pH 7.4)	g/ml	1.00	Lit.	1.00	[2]	1.00	Lit.	1.00	[2]	Solubility
Solubility succ. (pH 5.5)	g/ml	0.16	Lit.	0.16	[6]	0.16	Lit.	0.16	[6]	Solubility
logP	-	1.77	Lit.	1.77	[57]	1.77	Lit.	1.77	[57]	Lipophilicity
f_u	%	88	Lit.	88	[32]	88	Lit.	88	[32]	Fraction unbound
CYP2D6 $K_m \rightarrow \alpha$HM	µmol/l	10.08	Lit.	10.08‡	[33]	10.75	Lit.	10.75‡	[33]	Michaelis-Menten constant
CYP2D6 $k_{cat}{}^{NM} \rightarrow \alpha$HM	1/min	6.02	Optim.†	7.50	[33]	6.55	Optim.†	8.27	[33]	Catalytic rate constant
CYP2D6 $k_{cat}{}^{AS=2} \rightarrow \alpha$HM	1/min	10.17	Optim.†	-	-	11.19	Optim.†	-	-	Catalytic rate constant
CYP2D6 $K_m \rightarrow$ ODM	µmol/l	8.82	Lit.	8.82‡	[33]	12.43	Lit.	12.43‡	[33]	Michaelis-Menten constant
CYP2D6 $k_{cat}{}^{NM} \rightarrow$ ODM	1/min	9.87	Optim.†	12.30	[33]	8.21	Optim.†	10.37	[33]	Catalytic rate constant
CYP2D6 $k_{cat}{}^{AS=2} \rightarrow$ ODM	1/min	16.69	Optim.†	-	-	14.02	Optim.†	-	-	Catalytic rate constant
$CL_{hep,\ unsp.}$	1/min	0.08	Optim.	-	-	0.09	Optim.	-	-	Unspecific hepatic clearance
GFR fraction	-	1.00	Asm.	-	-	1.00	Asm.	-	-	Filtered drug in the urine
EHC continuous fraction	-	1.00	Asm.	-	-	1.00	Asm.	-	-	Bile fraction cont. released
NR Weibull time parameter	min	12.31	Optim.	-	[20, 23]	12.31	Optim.	-	[20, 23]	Dissolution profile time
NR Weibull shape parameter	-	0.72	Optim.	-	[20, 23]	0.72	Optim.	-	[20, 23]	Dissolution profile shape
CR Weibull time parameter	min	331.92	Optim.	-	[11]	331.92	Optim.	-	[11]	Dissolution profile time
CR Weibull shape parameter	-	1.53	Optim.	-	[11]	1.53	Optim.	-	[11]	Dissolution profile shape
Partition coefficients	-	Diverse	Calc.	R&R	[43, 44]	Diverse	Calc.	R&R	[43, 44]	Cell to plasma partitioning
Cellular permeability	cm/min	4.64E-03	Calc.	PK-Sim	[37]	4.64E-03	Calc.	PK-Sim	[37]	Perm. into cellular space
Intestinal permeability	cm/min	4.14E-05	Optim.	1.12E-05	Calc. [50]	4.14E-05	Optim.	1.12E-05	Calc. [50]	Transcellular intestinal perm.

-: not available, †: all CYP2D6 k_{cat} values were optimized in a fixed ratio ($k_{cat} \rightarrow \alpha$HM:$k_{cat} \rightarrow$ ODM) equivalent to the ratio of reported v_{max} values [33].
‡: in vitro values corrected for binding in the assay using estimated fraction unbound to microsomal protein ($fu_{mic,\ estimated}$ = 84%) [1], αHM: α-hydroxylation,
asm.: assumed, CR: controlled release release tablet, calc.: calculated, cont.: continously, CYP2D6: cytochrome P450 2D6, EHC: enterohepatic circulation, lit.: literature,
GFR: glomerular filtration rate, NR: normal release tablet, NM: normal metabolizer, ODM: *O*-demethylation, optim.: optimized, PK-Sim: PK-Sim calculation method,
R&R: Rodgers and Rowland calculation method, succ.: metoprolol succinate, tart.: metoprolol tartrate, unsp.: unspecific

S2.4 Drug-dependent parameters: α-hydroxymetoprolol

Table S2.4.3: α-hydroxymetoprolol drug-dependent parameters

Parameter	Unit	Value	Source	Literature	Reference	Description
MW	g/mol	283.36	Lit.	283.36	[24]	Molecular weight
pKa (strongest basic)	-	9.67	Lit.	9.67	[55]	Acid dissociation constant
pKa (strongest acidic)	-	13.55	Lit.	13.55	[55]	Acid dissociation constant
Solubility	g/ml	1.43	Lit.	1.43	[55]	Solubility
logP	-	0.87	Optim.	0.84	[55]	Lipophilicity
f_u	%	63	Calc.	63	[53]	Fraction unbound
$CL_{hep, unsp.}$	1/min	0.34	Optim.	-	-	Unspecific hepatic clearance
GFR fraction	-	1.00	Asm.	-	-	Filtered drug in the urine
EHC continuous fraction	-	1.00	Asm.	-	-	Bile fraction cont. released
Partition coefficients	-	Diverse	Calc.	R&R	[43, 44]	Cell to plasma partitioning
Cellular permeability	cm/min	4.08E-04	Calc.	PK-Sim	[37]	Perm. into the cellular space
Intestinal permeability	cm/min	1.08E-06	Calc.	1.08E-06	Calc. [50]	Transcellular intestinal perm.

-: not available, calc.: calculated, cont.: continuously, EHC: enterohepatic circulation, intest.: intestinal, GFR: glomerular filtration rate, perm.: permeability, PK-Sim: PK-Sim calculation method, R&R: Rodgers and Rowland calculation method, unsp.: unspecific

S2.5 Plasma profiles

S2.5.1 Semilogarithmic plots

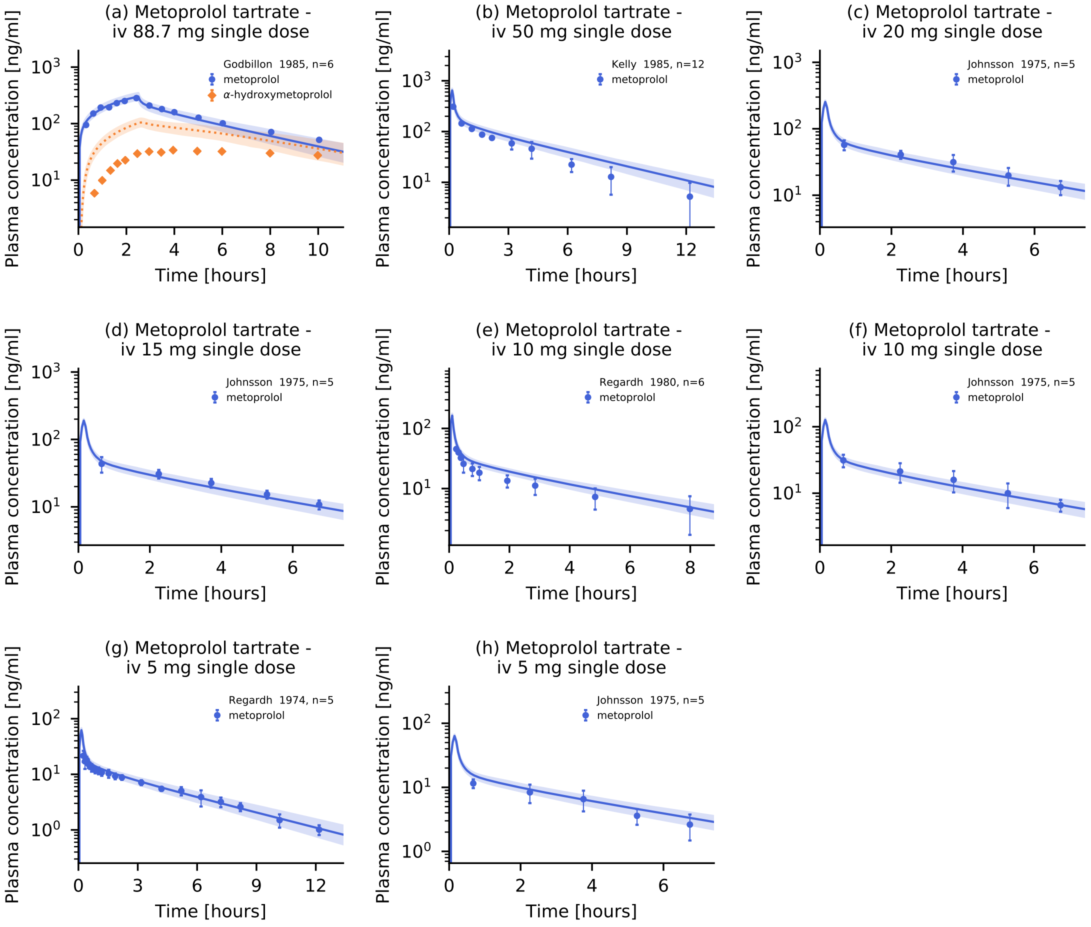

Figure S2.5.1: Metoprolol plasma concentrations. Model predictions of metoprolol and its metabolite *α*-hydroxymetoprolol plasma concentration-time profiles of intravenous studies of the training and test datasets, compared to observed data (semilogarithmic representation). Population predictions (n=100) are shown as lines with ribbons (arithmetic mean ± standard deviation (SD)), symbols represent the corresponding observed data ± SD. iv: intravenous

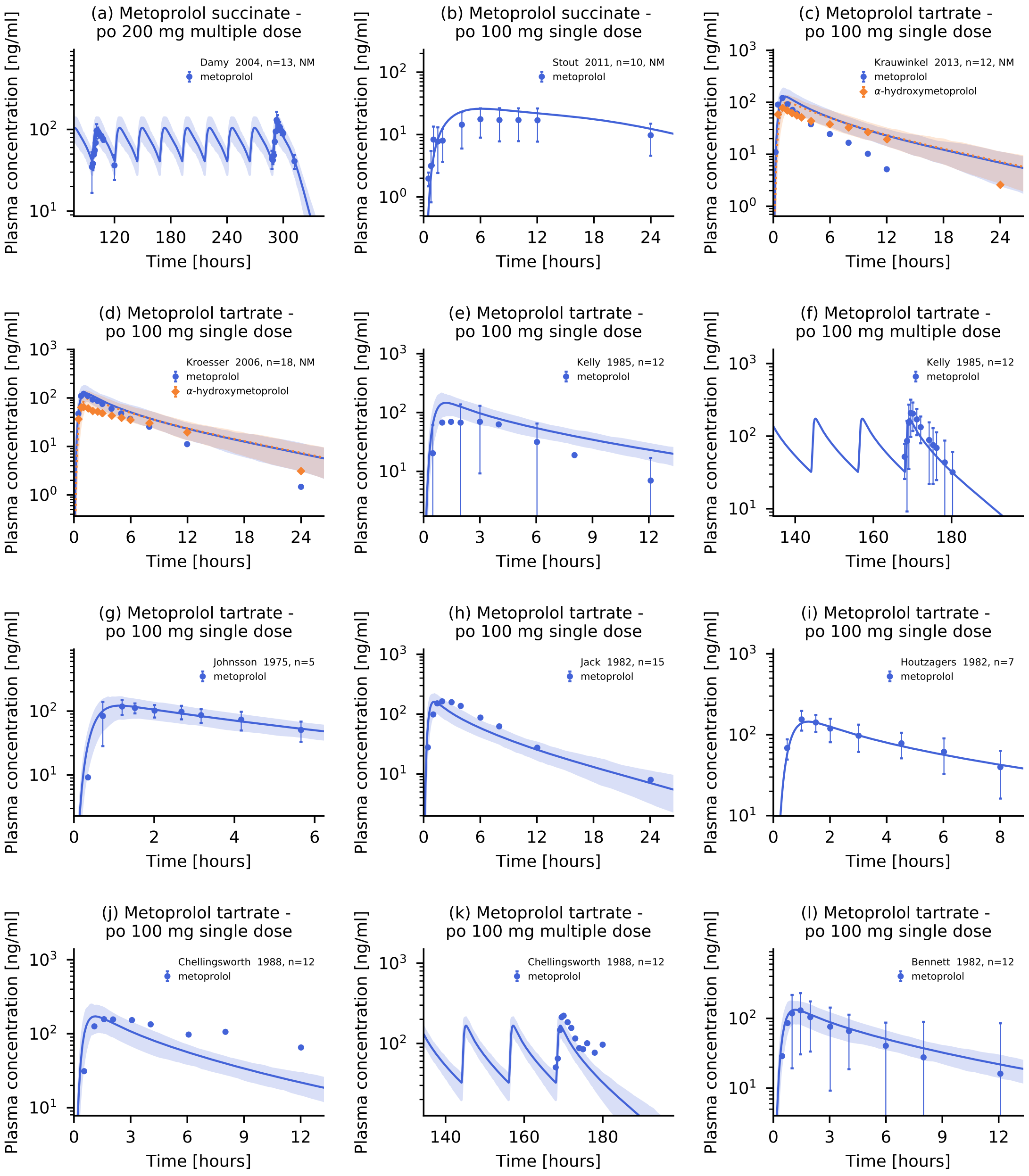

Figure S2.5.2: Metoprolol plasma concentrations. Model predictions of metoprolol and its metabolite *α*-hydroxymetoprolol plasma concentration-time profiles of oral studies of the training and test datasets, compared to observed data (semilogarithmic representation). Population predictions (n=100) are shown as lines with ribbons (arithmetic mean ± standard deviation (SD)), symbols represent the corresponding observed data ± SD. NM: normal metabolizer, po: oral

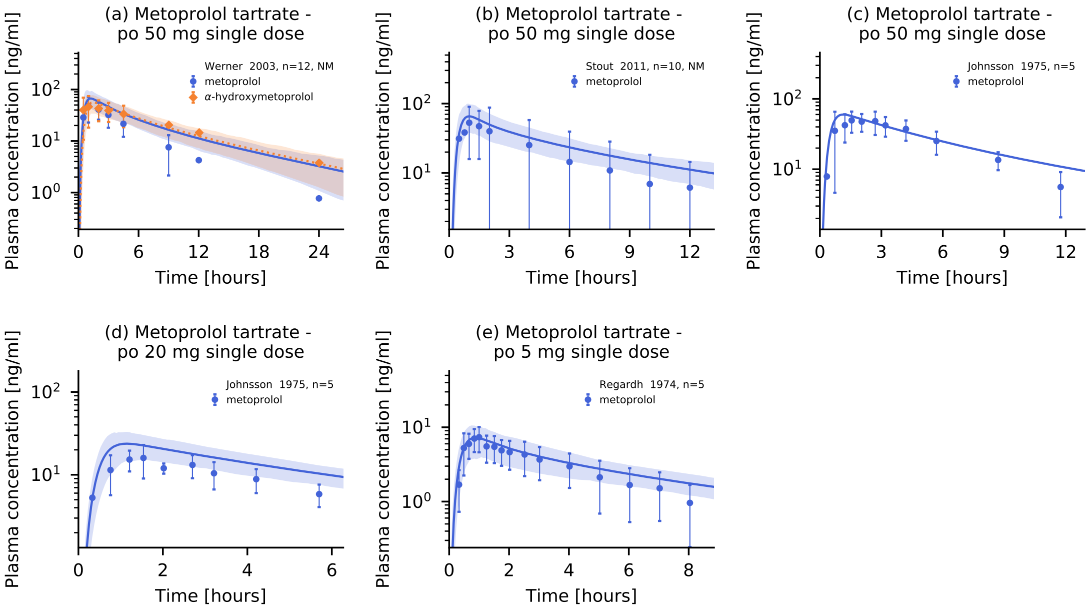

Figure S2.5.3: Metoprolol plasma concentrations. Model predictions of metoprolol and its metabolite α-hydroxymetoprolol plasma concentration-time profiles of oral studies of the training and test datasets, compared to observed data (semilogarithmic representation). Population predictions (n=100) are shown as lines with ribbons (arithmetic mean $\pm$ standard deviation (SD)), symbols represent the corresponding observed data $\pm$ SD. NM: normal metabolizer, po: oral

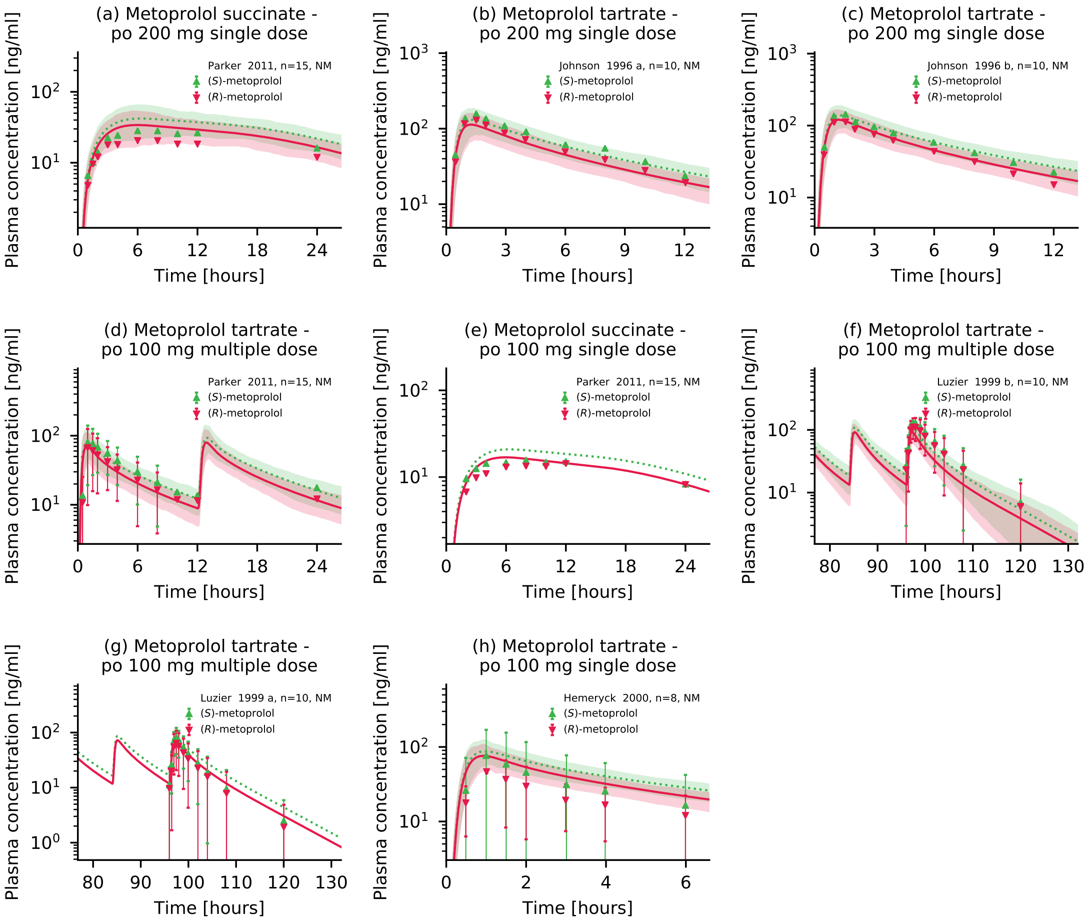

Figure S2.5.4: Metoprolol enantiomers plasma concentrations. Model predictions of *(R)*-metoprolol and *(S)*-metoprolol plasma concentration-time profiles of oral studies of the training and test datasets, compared to observed data (semilogarithmic representation). Population predictions (n=100) are shown as lines with ribbons (arithmetic mean $\pm$ standard deviation (SD)), symbols represent the corresponding observed data $\pm$ SD. NM: normal metabolizer, po: oral

S2.5.2 Linear plots

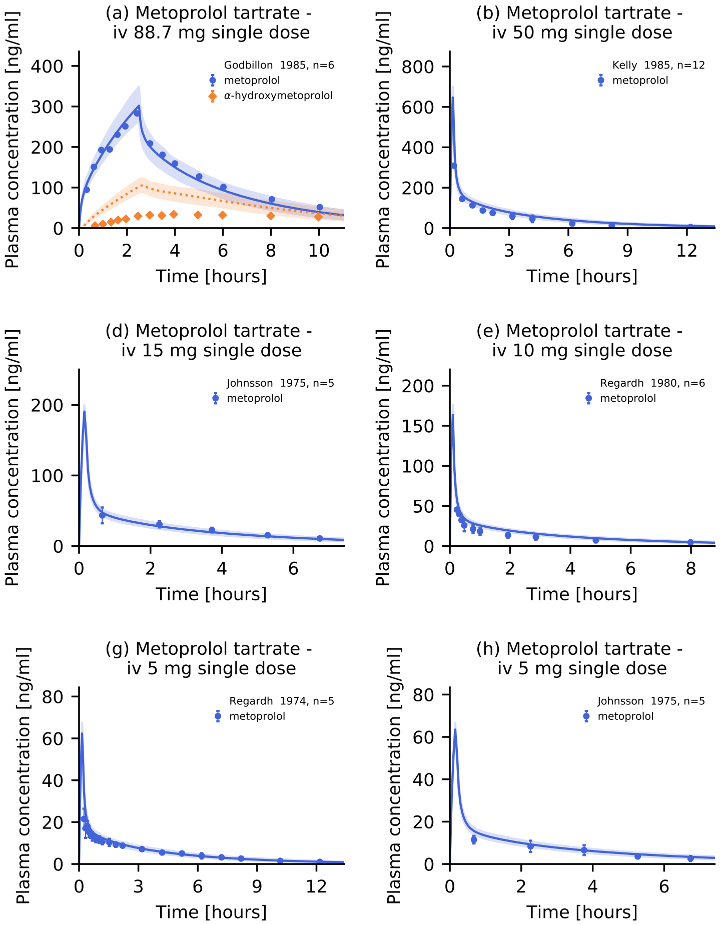

Figure S2.5.5: Metoprolol plasma concentrations. Model predictions of metoprolol and its metabolite *α*-hydroxymetoprolol plasma concentration-time profiles of intravenous studies of the training and test datasets, compared to observed data (linear representation). Population predictions (n=100) are shown as lines with ribbons (arithmetic mean ± standard deviation (SD)), symbols represent the corresponding observed data ± SD. iv: intravenous

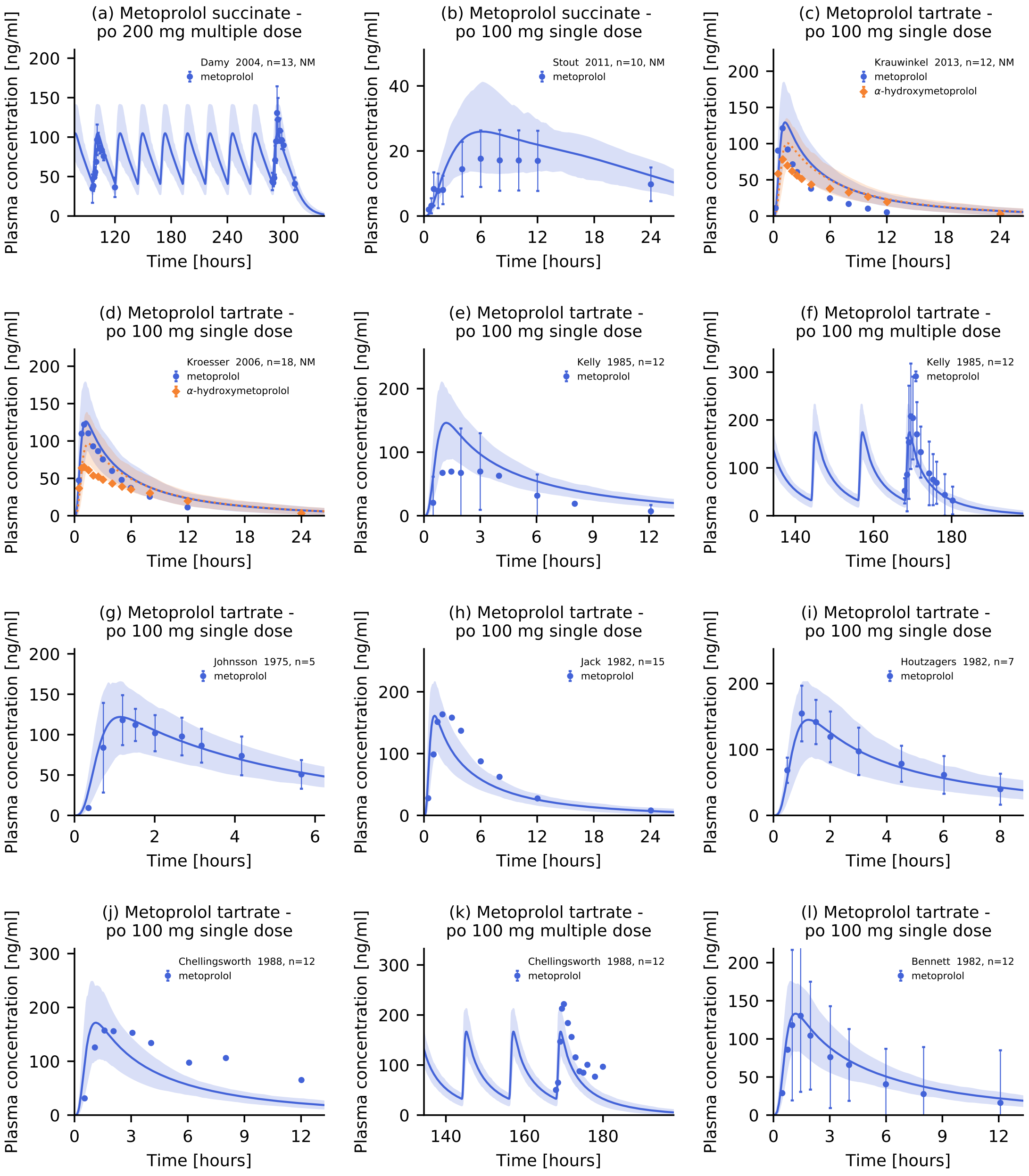

Figure S2.5.6: Metoprolol plasma concentrations. Model predictions of metoprolol and its metabolite α-hydroxymetoprolol plasma concentration-time profiles of oral studies of the training and test datasets, compared to observed data (linear representation). Population predictions (n=100) are shown as lines with ribbons (arithmetic mean ± standard deviation (SD)), symbols represent the corresponding observed data ± SD. NM: normal metabolizer, po: oral

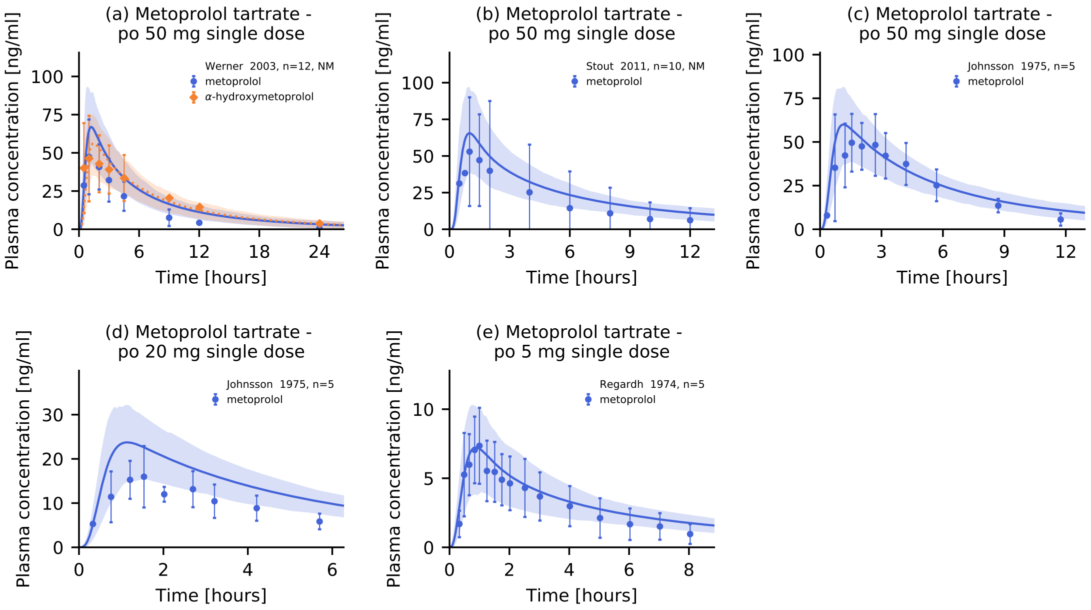

Figure S2.5.7: Metoprolol plasma concentrations. Model predictions of metoprolol and its metabolite α-hydroxymetoprolol plasma concentration-time profiles of oral studies of the training and test datasets, compared to observed data (linear representation). Population predictions (n=100) are shown as lines with ribbons (arithmetic mean $\pm$ standard deviation (SD)), symbols represent the corresponding observed data $\pm$ SD. NM: normal metabolizer, po: oral

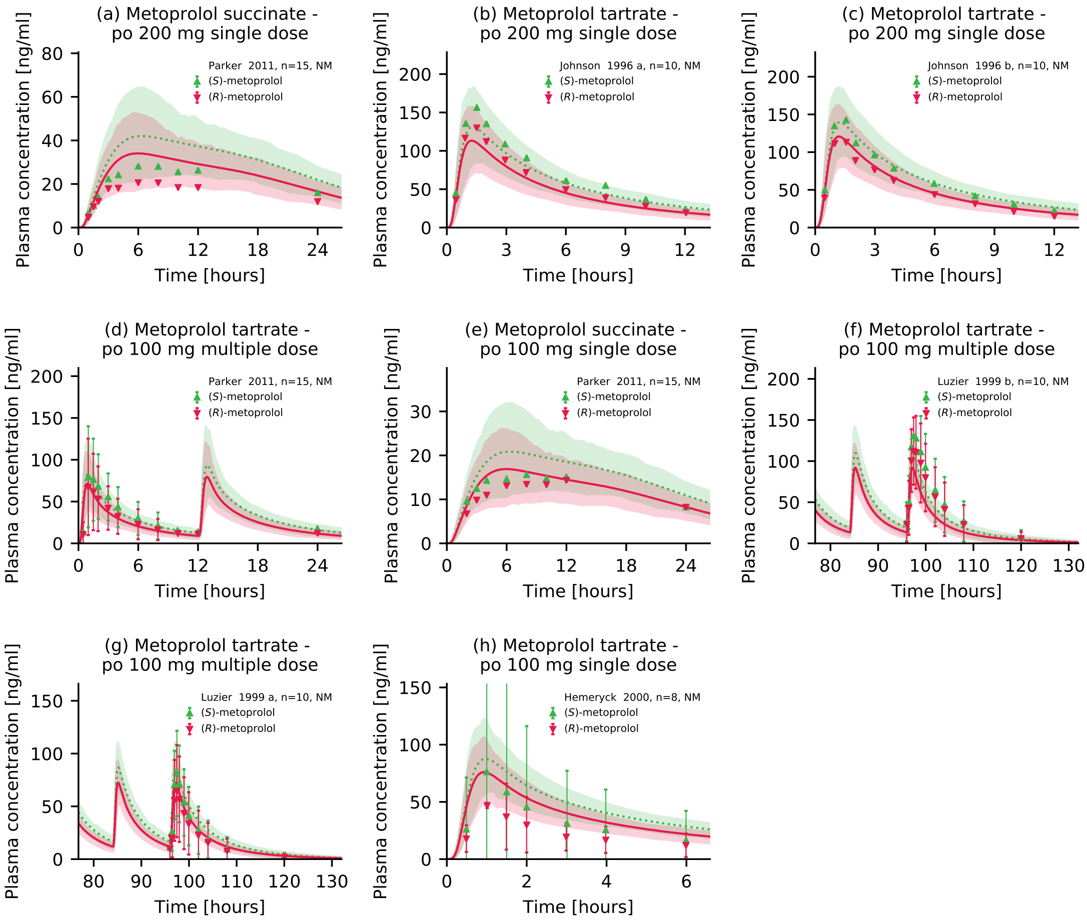

Figure S2.5.8: Metoprolol enantiomers plasma concentrations. Model predictions of *(R)*-metoprolol and *(S)*-metoprolol plasma concentration-time profiles of oral studies of the training and test datasets, compared to observed data (linear representation). Population predictions (n=100) are shown as lines with ribbons (arithmetic mean ± standard deviation (SD)), symbols represent the corresponding observed data ± SD. NM: normal metabolizer, po: oral

S2.6 Model evaluation

S2.6.1 Plasma concentrations goodness-of-fit plots

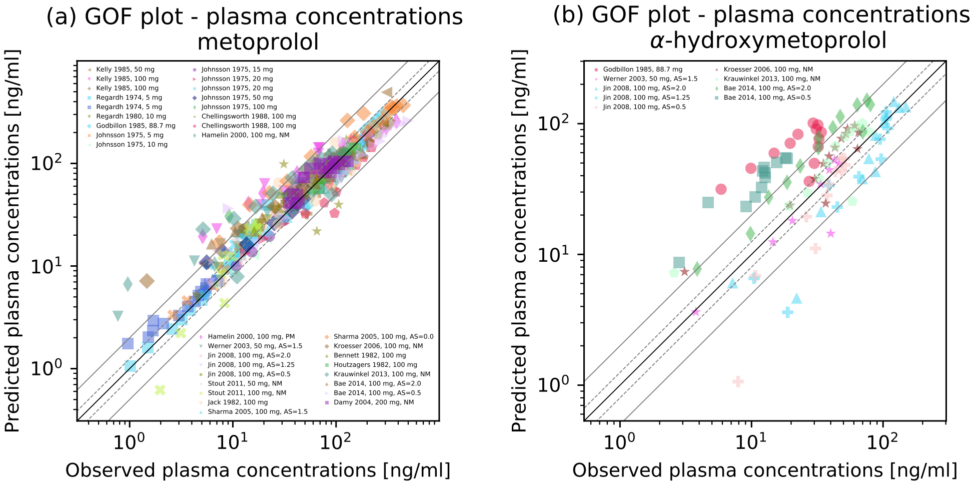

Figure S2.6.9: Plasma concentrations goodness-of-fit plots of the final metoprolol model. Predicted versus observed plasma concentrations for (a) metoprolol and (b) α-hydroxymetoprolol for all studies. The solid black line indicates the line of identity, solid grey lines show 2-fold deviation, dashed grey lines indicate 1.25-fold deviation. AS: CYP2D6 activity score, gof: goodness-of-fit, NM: normal metabolizer, PM: poor metabolizer, vs: versus

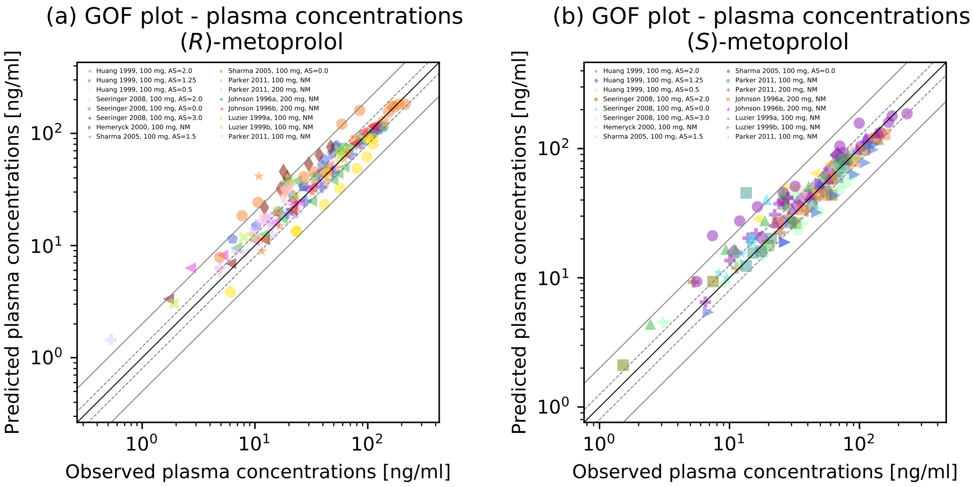

Figure S2.6.10: Plasma concentrations goodness-of-fit plots of the final metoprolol model. Predicted versus observed plasma concentrations for (a) *(R)*-metoprolol and (b) *(S)*-metoprolol for all studies. The solid black line indicates the line of identity, solid grey lines show 2-fold deviation, dashed grey lines indicate 1.25-fold deviation. AS: CYP2D6 activity score, gof: goodness-of-fit, NM: normal metabolizer, vs: versus

S2.6.2 Mean relative deviation of plasma concentration predictions (metoprolol, α-hydroxymetoprolol)

Table S2.6.4: Mean relative deviation of plasma concentration predictions (metoprolol, α-hydroxymetoprolol)

Dosing	Molecule	CYP2D6 status	MRD	Reference
iv, inf, 88.7 mg	α-hydroxymetoprolol	-	3.12	Godbillon et al. 1985 [12]
po, tab, 100 mg	α-hydroxymetoprolol	AS=2.0	1.99	Bae et al. 2014 [3]
po, tab, 100 mg	α-hydroxymetoprolol	AS=0.5	3.25	Bae et al. 2014 [3]
po, tab, 100 mg	α-hydroxymetoprolol	AS=2.0	1.79	Jin et al. 2008 [19]
po, tab, 100 mg	α-hydroxymetoprolol	AS=1.25	1.85	Jin et al. 2008 [19]
po, tab, 100 mg	α-hydroxymetoprolol	AS=0.5	2.02	Jin et al. 2008 [19]
po, -, 100 mg	α-hydroxymetoprolol	NM	1.62	Krauwinkel et al. 2013 [26]
po, tab, 100 mg	α-hydroxymetoprolol	NM	1.54	Kroesser et al. 2006 [27]
po, tab, 50 mg	α-hydroxymetoprolol	AS=1.5	1.46	Werner et al. 2003 [54]
iv, inf, 88.7 mg	metoprolol	-	1.11	Godbillon et al. 1985 [12]
iv, inf, 50 mg	metoprolol	-	1.51	Kelly et al. 1985 [23]
iv, inf, 20 mg	metoprolol	-	1.11	Johnsson et al. 1975 [22]
iv, inf, 15 mg	metoprolol	-	1.11	Johnsson et al. 1975 [22]
iv, inf, 10 mg	metoprolol	-	1.13	Johnsson et al. 1975 [22]
iv, inf, 10 mg	metoprolol	-	1.31	Regardh et al. 1980 [42]
iv, inf, 5 mg	metoprolol	-	1.23	Johnsson et al. 1975 [22]
iv, inf, 5 mg	metoprolol	-	1.20	Regardh et al. 1974 [41]
po, CR, tab, 200 mg, daily	metoprolol	NM	1.25	Damy et al. 2004 [11]
po, tab, 100 mg	metoprolol	AS=2.0	1.53	Bae et al. 2014 [3]
po, tab, 100 mg	metoprolol	AS=0.5	1.77	Bae et al. 2014 [3]
po, -, 100 mg	metoprolol	-	1.29	Bennett et al. 1982 [4]
po, tab, 100 mg	metoprolol	-	1.67	Chellingsworth et al. 1988 [9]
po, tab, 100 mg, bid	metoprolol	-	1.99	Chellingsworth et al. 1988 [9]
po, tab, 100 mg	metoprolol	NM	1.95	Hamelin et al. 2000 [14]
po, tab, 100 mg	metoprolol	PM	1.90	Hamelin et al. 2000 [14]
po, tab, 100 mg	metoprolol	-	1.09	Houtzagers et al. 1982 [16]
po, tab, 100 mg	metoprolol	-	1.51	Jack et al. 1982 [18]
po, tab, 100 mg	metoprolol	AS=2.0	1.24	Jin et al. 2008 [19]
po, tab, 100 mg	metoprolol	AS=1.25	1.24	Jin et al. 2008 [19]
po, tab, 100 mg	metoprolol	AS=0.5	1.34	Jin et al. 2008 [19]
po, tab, 100 mg	metoprolol	-	1.50	Johnsson et al. 1975 [22]
po, -, 100 mg, bid	metoprolol	-	1.33	Kelly et al. 1985 [23]
po, -, 100 mg	metoprolol	-	2.13	Kelly et al. 1985 [23]
po, -, 100 mg	metoprolol	NM	2.06	Krauwinkel et al. 2013 [26]
po, tab, 100 mg	metoprolol	NM	1.68	Kroesser et al. 2006 [27]
po, -, 100 mg	metoprolol	AS=1.5	1.26	Sharma et al. 2005 [47]
po, -, 100 mg	metoprolol	AS=0.0	1.67	Sharma et al. 2005 [47]
po, CR, tab, 100 mg	metoprolol	NM	1.66	Stout et al. 2011 [48]

-: not available, AS: CYP2D6 activity score, bid: twice daily, CR: controlled release, inf: infusion, iv: intravenous NM: normal metabolizer, PM: poor metabolizer, po: oral,sol: oral solution, tab: tablet

Table S2.6.4: Mean relative deviation of plasma concentration predictions (metoprolol, α-hydroxymetoprolol)

Dosing	Molecule	CYP2D6 status	MRD	Reference
po, tab, 50 mg	metoprolol	-	1.37	Johnsson et al. 1975 [22]
po, tab, 50 mg	metoprolol	NM	1.52	Stout et al. 2011 [48]
po, tab, 50 mg	metoprolol	AS=1.5	2.06	Werner et al. 2003 [54]
po, tab, 20 mg	metoprolol	-	1.55	Johnsson et al. 1975 [22]
po, sol, 5 mg	metoprolol	-	1.29	Regardh et al. 1974 [41]
MRD			1.61 (1.09–3.25)<br>37/43 with MRD ≤ 2	

-: not available, AS: CYP2D6 activity score, bid: twice daily, CR: controlled release, inf: infusion, iv: intravenous NM: normal metabolizer, PM: poor metabolizer, po: oral,sol: oral solution, tab: tablet

S2.6.3 Mean relative deviation of plasma concentration predictions ((*R*)-metoprolol, (*S*)-metoprolol)

Table S2.6.5: Mean relative deviation of plasma concentration predictions ((*R*)-metoprolol, (*S*)-metoprolol)

Dosing	Molecule	CYP2D6 status	MRD	Reference
po, tab, 200 mg	(*R*)-metoprolol	NM	1.13	Johnson et al. 1996 a [20]
po, tab, 200 mg	(*R*)-metoprolol	NM	1.13	Johnson et al. 1996 b [21]
po, CR, tab, 200 mg	(*R*)-metoprolol	NM	1.50	Parker et al. 2011 [39]
po, tab, 100 mg	(*R*)-metoprolol	NM	1.93	Hemeryck et al. 2000 [15]
po, tab, 100 mg	(*R*)-metoprolol	AS=2.0	1.34	Huang et al. 1999 [17]
po, tab, 100 mg	(*R*)-metoprolol	AS=1.25	1.39	Huang et al. 1999 [17]
po, tab, 100 mg	(*R*)-metoprolol	AS=0.5	1.24	Huang et al. 1999 [17]
po, tab, 100 mg, bid	(*R*)-metoprolol	NM	1.36	Luzier et al. 1999 a [30]
po, tab, 100 mg, bid	(*R*)-metoprolol	NM	1.55	Luzier et al. 1999 b [31]
po, tab, 100 mg, bid	(*R*)-metoprolol	NM	1.52	Parker et al. 2011 [39]
po, CR, tab, 100 mg	(*R*)-metoprolol	NM	1.24	Parker et al. 2011 [39]
po, tab, 100 mg	(*R*)-metoprolol	AS=3.0	1.41	Seeringer et al. 2008 [46]
po, tab, 100 mg	(*R*)-metoprolol	AS=2.0	1.62	Seeringer et al. 2008 [46]
po, tab, 100 mg	(*R*)-metoprolol	AS=0.0	1.37	Seeringer et al. 2008 [46]
po, -, 100 mg	(*R*)-metoprolol	AS=1.5	1.28	Sharma et al. 2005 [47]
po, -, 100 mg	(*R*)-metoprolol	AS=0.0	1.55	Sharma et al. 2005 [47]
po, tab, 200 mg	(*S*)-metoprolol	NM	1.14	Johnson et al. 1996 a [20]
po, tab, 200 mg	(*S*)-metoprolol	NM	1.09	Johnson et al. 1996 b [21]
po, CR, tab, 200 mg	(*S*)-metoprolol	NM	1.42	Parker et al. 2011 [39]
po, tab, 100 mg	(*S*)-metoprolol	NM	1.56	Hemeryck et al. 2000 [15]
po, tab, 100 mg	(*S*)-metoprolol	AS=2.0	1.20	Huang et al. 1999 [17]
po, tab, 100 mg	(*S*)-metoprolol	AS=1.25	1.20	Huang et al. 1999 [17]
po, tab, 100 mg	(*S*)-metoprolol	AS=0.5	1.17	Huang et al. 1999 [17]
po, tab, 100 mg, bid	(*S*)-metoprolol	NM	1.35	Luzier et al. 1999 a [30]
po, tab, 100 mg, bid	(*S*)-metoprolol	NM	1.44	Luzier et al. 1999 b [31]
po, tab, 100 mg, bid	(*S*)-metoprolol	NM	1.46	Parker et al. 2011 [39]
po, CR, tab, 100 mg	(*S*)-metoprolol	NM	1.29	Parker et al. 2011 [39]
po, tab, 100 mg	(*S*)-metoprolol	AS=3.0	1.31	Seeringer et al. 2008 [46]
po, tab, 100 mg	(*S*)-metoprolol	AS=2.0	1.29	Seeringer et al. 2008 [46]
po, tab, 100 mg	(*S*)-metoprolol	AS=0.0	1.43	Seeringer et al. 2008 [46]
po, -, 100 mg	(*S*)-metoprolol	AS=1.5	1.31	Sharma et al. 2005 [47]
po, -, 100 mg	(*S*)-metoprolol	AS=0.0	1.64	Sharma et al. 2005 [47]
MRD			1.37 (1.09–1.93)<br>32/32 with MRD ≤ 2	
Overall MRD (all four compounds)			1.51 (1.09–3.25)<br>69/75 with MRD ≤ 2	

-: not available, AS: CYP2D6 activity score, bid: twice daily, CR: controlled release, po: oral, tab: tablet.

S2.6.4 AUC$_{last}$ and C$_{max}$ values goodness-of-fit plots

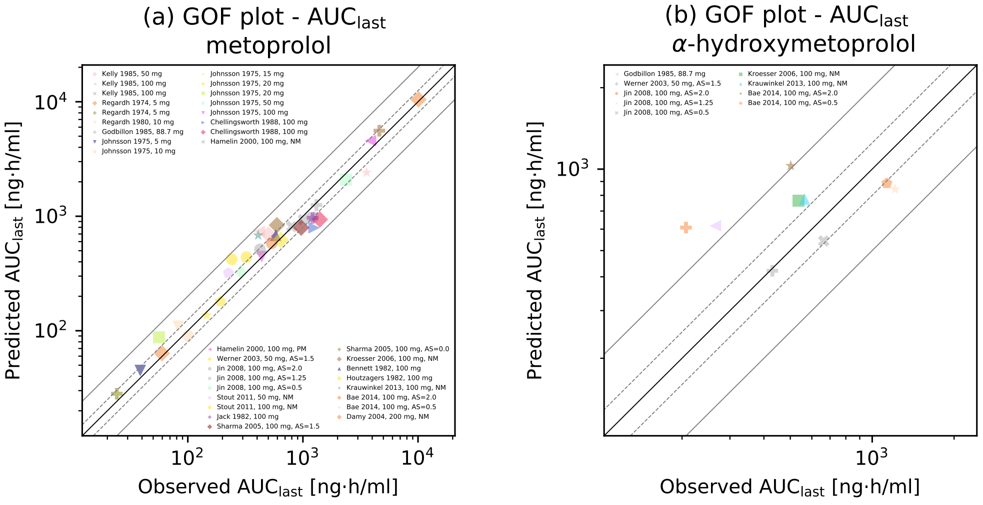

Figure S2.6.11: AUC$_{last}$ values goodness-of-fit plots for the final metoprolol model. Predicted versus observed AUC$_{last}$ values for racemic (a) metoprolol and (b) α-hydroxymetoprolol for all studies. The solid black line marks the line of identity, the dotted grey lines mark the 0.8- to 1.25-fold range, the dashed black lines indicate the 0.5- to 2-fold range. AS: CYP2D6 activity score, gof: goodness-of-fit, NM: normal metabolizer, PM: poor metabolizer, vs: versus

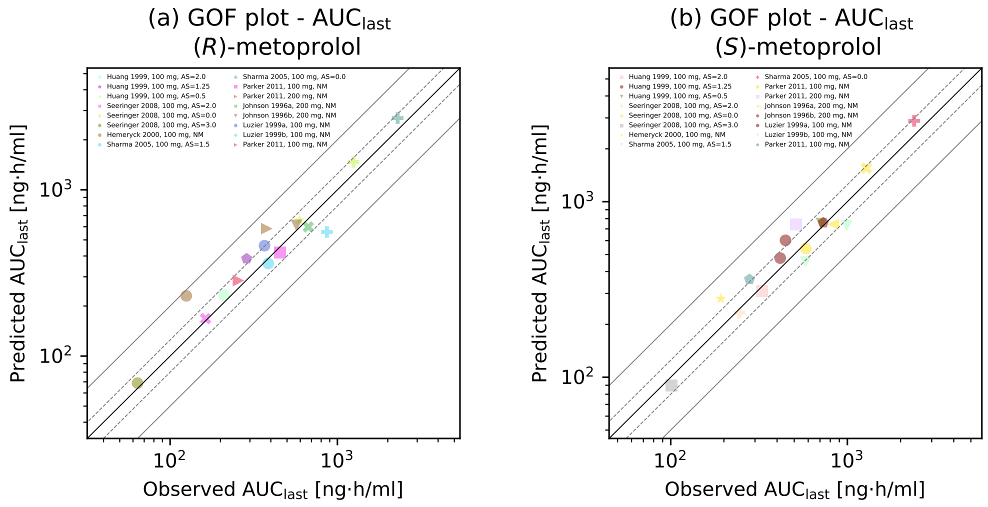

Figure S2.6.12: AUC_{last} goodness-of-fit plots for the final metoprolol model. Predicted versus observed AUC_{last} values for (a) (*S*)-metoprolol and (b) (*R*)-metoprolol for all studies. The solid black line marks the line of identity, the dotted grey lines mark the 0.8- to 1.25-fold range, the dashed black lines indicate the 0.5- to 2-fold range. AS: CYP2D6 activity score, gof: goodness-of-fit, NM: normal metabolizer, vs: versus

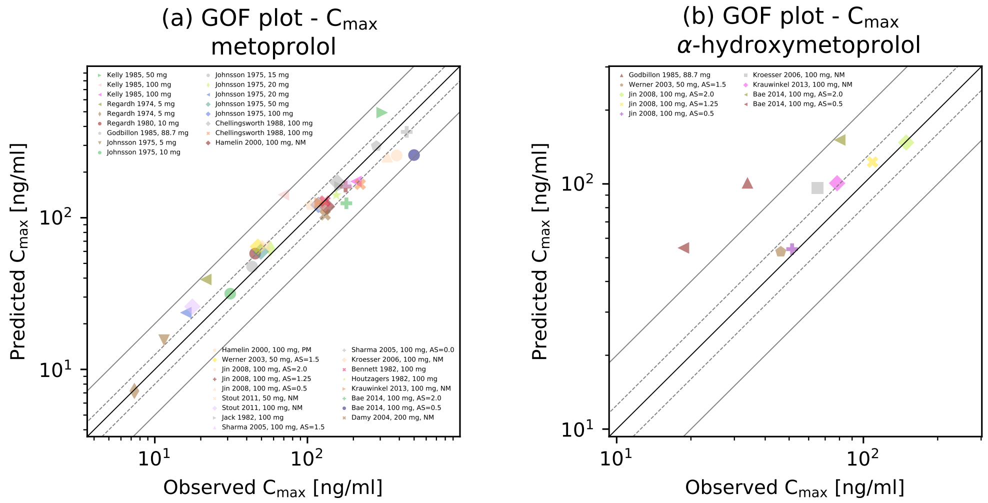

Figure S2.6.13: C_{max} values goodness-of-fit plots for the final metoprolol model. Predicted versus observed C_{max} values for racemic (a) metoprolol and (b) *α*-hydroxymetoprolol for all studies. The solid black line marks the line of identity, the dotted grey lines mark the 0.8- to 1.25-fold range, the dashed black lines indicate the 0.5- to 2-fold range. AS: CYP2D6 activity score, gof: goodness-of-fit, NM: normal metabolizer, PM: poor metabolizer, vs: versus

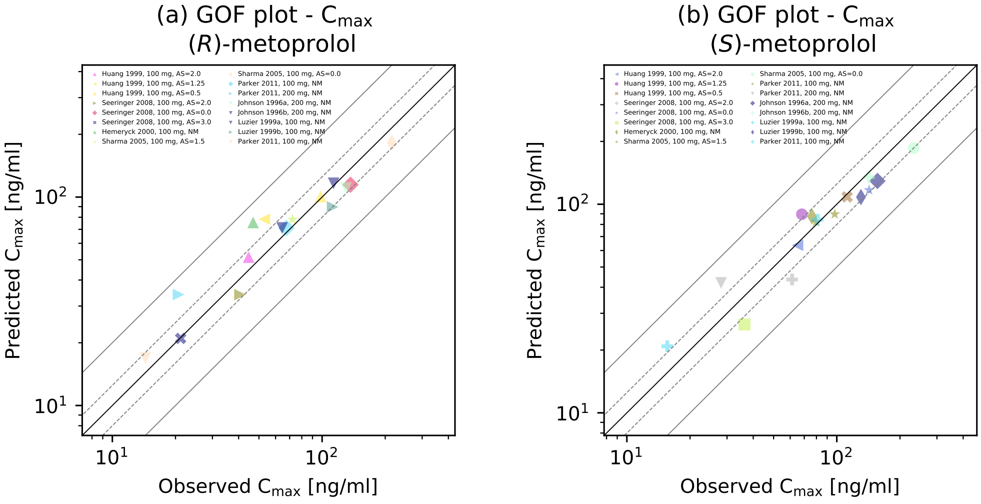

Figure S2.6.14: AUC_{last} goodness-of-fit plots for the final metoprolol model. Predicted versus observed AUC_{last} values for (a) (*S*)-metoprolol and (b) (*R*)-metoprolol for all studies. The solid black line marks the line of identity, the dotted grey lines mark the 0.8- to 1.25-fold range, the dashed black lines indicate the 0.5- to 2-fold range. AS: CYP2D6 activity score, gof: goodness-of-fit, NM: normal metabolizer, vs: versus

S2.6.5 Geometric mean fold error of predicted AUC_{last} and C_{max} values (metoprolol, α-hydroxymetoprolol)

Table S2.6.6: Predicted and observed AUC_{last} and C_{max} values (metoprolol, α-hydroxymetoprolol)

Dosing	Molecule	CYP2D6 status	AUC_{last} Pred [h·ng/ml]	AUC_{last} Obs [h·ng/ml]	AUC_{last} Pred/Obs	C_{max} Pred [ng/ml]	C_{max} Obs [ng/ml]	C_{max} Pred/Obs	Reference
iv, inf, 88.7 mg	α-hydroxymetoprolol	-	617.20	265.13	2.33	-	-	-	Godbillon et al. 1985 [12]
po, tab, 100 mg	α-hydroxymetoprolol	AS=2.0	1028.84	501.89	2.05	152.15	80.13	1.90	Bae et al. 2014 [3]
po, tab, 100 mg	α-hydroxymetoprolol	AS=0.5	607.99	206.78	2.94	55.34	18.64	2.97	Bae et al. 2014 [3]
po, tab, 100 mg	α-hydroxymetoprolol	AS=2.0	886.17	1131.93	0.78	148.83	149.04	1.00	Jin et al. 2008 [19]
po, tab, 100 mg	α-hydroxymetoprolol	AS=1.25	843.16	1212.70	0.70	123.06	108.88	1.13	Jin et al. 2008 [19]
po, tab, 100 mg	α-hydroxymetoprolol	AS=0.5	542.62	662.97	0.82	54.84	51.41	1.07	Jin et al. 2008 [19]
po, -, 100 mg	α-hydroxymetoprolol	NM	775.17	564.03	1.37	100.67	78.25	1.29	Krauwinkel et al. 2013 [26]
po, tab, 100 mg	α-hydroxymetoprolol	NM	765.96	534.92	1.43	96.50	65.12	1.48	Kroesser et al. 2006 [27]
po, tab, 50 mg	α-hydroxymetoprolol	AS=1.5	421.10	429.62	0.98	56.25	46.25	1.22	Werner et al. 2003 [54]
iv, inf, 88.7 mg	metoprolol	-	1251.70	1310.03	0.96	-	-	-	Godbillon et al. 1985 [12]
iv, inf, 50 mg	metoprolol	-	692.12	501.71	1.38	-	-	-	Kelly et al. 1985 [23]
iv, inf, 20 mg	metoprolol	-	179.84	192.31	0.94	-	-	-	Johnsson et al. 1975 [22]
iv, inf, 15 mg	metoprolol	-	136.42	145.74	0.94	-	-	-	Johnsson et al. 1975 [22]
iv, inf, 10 mg	metoprolol	-	90.64	100.45	0.90	-	-	-	Johnsson et al. 1975 [22]
iv, inf, 10 mg	metoprolol	-	109.25	82.28	1.33	-	-	-	Regardh et al. 1980 [42]
iv, inf, 5 mg	metoprolol	-	44.91	38.63	1.16	-	-	-	Johnsson et al. 1975 [22]
iv, inf, 5 mg	metoprolol	-	63.61	59.56	1.07	-	-	-	Regardh et al. 1974 [41]
po, CR, tab, 200 mg, daily	metoprolol	NM	10470.95	10087.41	1.04	104.92	130.54	0.80	Damy et al. 2004 [11]
po, tab, 100 mg	metoprolol	AS=2.0	580.39	535.59	1.08	126.63	179.95	0.70	Bae et al. 2014 [3]
po, tab, 100 mg	metoprolol	AS=0.5	2410.05	3570.91	0.67	259.49	499.36	0.52	Bae et al. 2014 [3]
po, -, 100 mg	metoprolol	-	682.28	580.40	1.18	133.15	130.30	1.02	Bennett et al. 1982 [4]
po, tab, 100 mg bid	metoprolol	-	936.86	1401.85	0.67	166.91	221.75	0.75	Chellingsworth et al. 1988 [9]
po, tab, 100 mg	metoprolol	-	792.38	1250.28	0.63	171.58	157.08	1.09	Chellingsworth et al. 1988 [9]
po, tab, 100 mg	metoprolol	NM	812.74	798.21	1.02	132.33	134.26	0.99	Hamelin et al. 2000 [14]
po, tab, 100 mg	metoprolol	PM	4569.86	3861.13	1.18	266.39	384.09	0.69	Hamelin et al. 2000 [14]
po, tab, 100 mg	metoprolol	-	620.28	640.42	0.97	145.05	154.54	0.94	Houtzagers et al. 1982 [16]
po, tab, 100 mg	metoprolol	-	968.18	1213.24	0.80	161.20	163.49	0.99	Jack et al. 1982 [18]
po, tab, 100 mg	metoprolol	AS=2.0	514.07	423.56	1.21	122.15	104.49	1.17	Jin et al. 2008 [19]
po, tab, 100 mg	metoprolol	AS=1.25	934.51	1009.15	0.93	164.11	177.83	0.92	Jin et al. 2008 [19]
po, tab, 100 mg	metoprolol	AS=0.5	2095.14	2367.57	0.88	254.16	332.72	0.76	Jin et al. 2008 [19]
po, tab, 100 mg	metoprolol	-	444.47	437.96	1.01	121.90	117.92	1.03	Johnsson et al. 1975 [22]
po, -, 100 mg bid	metoprolol	-	980.20	1208.41	0.81	174.50	207.73	0.84	Kelly et al. 1985 [23]
po, -, 100 mg	metoprolol	-	737.05	416.18	1.77	146.25	69.54	2.10	Kelly et al. 1985 [23]
po, -, 100 mg	metoprolol	NM	681.74	408.75	1.67	129.35	121.20	1.07	Krauwinkel et al. 2013 [26]
po, tab, 100 mg	metoprolol	NM	835.96	594.21	1.41	126.23	121.97	1.03	Kroesser et al. 2006 [27]
po, -, 100 mg	metoprolol	AS=1.5	795.72	965.96	0.82	177.69	183.30	0.97	Sharma et al. 2005 [47]
po, -, 100 mg	metoprolol	AS=0.0	5585.63	4612.83	1.21	368.73	447.28	0.82	Sharma et al. 2005 [47]
po, CR, tab, 100 mg	metoprolol	NM	439.65	323.13	1.36	25.96	17.62	1.47	Stout et al. 2011 [48]
po, tab, 50 mg	metoprolol	-	323.70	289.99	1.12	59.99	49.54	1.21	Johnsson et al. 1975 [22]
po, tab, 50 mg	metoprolol	NM	319.14	225.06	1.42	65.57	52.93	1.24	Stout et al. 2011 [48]
po, tab, 50 mg	metoprolol	AS=1.5	421.32	240.97	1.75	66.95	47.34	1.41	Werner et al. 2003 [54]
po, tab, 20 mg	metoprolol	-	87.44	56.33	1.55	23.74	15.96	1.49	Johnsson et al. 1975 [22]
po, sol, 5 mg	metoprolol	-	28.19	24.18	1.17	7.23	7.35	0.98	Regardh et al. 1974 [41]
Overall GMFE					1.31 (1.01–2.94)			1.27 (1.00–2.97)	

-: not available, AUC_{last}: AUC from the time of the first concentration measurement to the last time point of concentration measurement, bid: twice daily, CR: controlled release, GMFE: geometric mean fold error, inf: infusion, iv: intravenous, po: oral, Pred: predicted, Obs: observed, sol: oral solution, tab: tablet

Table S2.6.6: Predicted and observed AUC_{last} and C_{max} values (metoprolol, α-hydroxymetoprolol) *(continued)*

Dosing	Molecule	CYP2D6 status	AUC_{last} Pred [h·ng/ml]	AUC_{last} Obs [h·ng/ml]	AUC_{last} Pred/Obs	C_{max} Pred [ng/ml]	C_{max} Obs [ng/ml]	C_{max} Pred/Obs	Reference
					40/43 with GMFE $\leq$ 2			32/34 with GMFE $\leq$ 2	

-: not available, AUC_{last}: AUC from the time of the first concentration measurement to the last time point of concentration measurement, bid: twice daily, CR: controlled release, GMFE: geometric mean fold error, inf: infusion, iv: intravenous, po: oral, Pred: predicted, Obs: observed, sol: oral solution, tab: tablet

S2.6.6 Geometric mean fold error of predicted AUC_{last} and C_{max} values ((*R*)-metoprolol, (*S*)-metoprolol)

Table S2.6.7: Predicted and observed AUC_{last} and C_{max} values ((*R*)-metoprolol, (*S*)-metoprolol)

Dosing	Molecule	CYP2D6 status	AUC_{last} Pred [h·ng/ml]	AUC_{last} Obs [h·ng/ml]	AUC_{last} Pred/Obs	C_{max} Pred [ng/ml]	C_{max} Obs [ng/ml]	C_{max} Pred/Obs	Reference
po, tab, 200 mg	(*R*)-metoprolol	NM	598.16	669.28	0.89	113.26	130.14	0.87	Johnson et al. 1996 a [20]
po, tab, 200 mg	(*R*)-metoprolol	NM	610.43	569.08	1.07	120.72	113.42	1.06	Johnson et al. 1996 b [21]
po, CR, tab, 200 mg	(*R*)-metoprolol	NM	584.15	378.38	1.54	34.06	20.67	1.65	Parker et al. 2011 [39]
po, tab, 100 mg	(*R*)-metoprolol	NM	230.19	124.91	1.84	76.01	46.90	1.62	Hemeryck et al. 2000 [15]
po, tab, 100 mg	(*R*)-metoprolol	AS=2.0	232.98	210.57	1.11	52.48	44.64	1.18	Huang et al. 1999 [17]
po, tab, 100 mg	(*R*)-metoprolol	AS=1.25	385.55	286.63	1.35	79.93	53.02	1.51	Huang et al. 1999 [17]
po, tab, 100 mg	(*R*)-metoprolol	AS=0.5	656.06	593.93	1.10	101.14	98.46	1.03	Huang et al. 1999 [17]
po, tab, 100 mg, bid	(*R*)-metoprolol	NM	461.34	366.57	1.26	72.56	64.46	1.13	Luzier et al. 1999 a [30]
po, tab, 100 mg, bid	(*R*)-metoprolol	NM	557.74	865.88	0.64	92.11	112.25	0.82	Luzier et al. 1999 b [31]
po, tab, 100 mg, bid	(*R*)-metoprolol	NM	418.97	453.99	0.92	79.58	67.56	1.18	Parker et al. 2011 [39]
po, CR, tab, 100 mg	(*R*)-metoprolol	NM	284.50	256.35	1.11	16.90	14.36	1.18	Parker et al. 2011 [39]
po, tab, 100 mg	(*R*)-metoprolol	AS=3.0	68.78	63.90	1.08	27.15	21.16	1.28	Seeringer et al. 2008 [46]
po, tab, 100 mg	(*R*)-metoprolol	AS=2.0	167.96	162.85	1.03	41.22	40.60	1.02	Seeringer et al. 2008 [46]
po, tab, 100 mg	(*R*)-metoprolol	AS=0.0	1471.47	1248.95	1.18	115.72	136.60	0.85	Seeringer et al. 2008 [46]
po, -, 100 mg	(*R*)-metoprolol	AS=1.5	360.85	385.76	0.94	82.29	72.24	1.14	Sharma et al. 2005 [47]
po, -, 100 mg	(*R*)-metoprolol	AS=0.0	2701.57	2291.56	1.18	182.75	215.01	0.85	Sharma et al. 2005 [47]
po, tab, 200 mg	(*S*)-metoprolol	NM	742.75	832.00	0.89	131.15	156.19	0.84	Johnson et al. 1996 a [20]
po, tab, 200 mg	(*S*)-metoprolol	NM	758.90	730.46	1.04	139.53	142.40	0.98	Johnson et al. 1996 b [21]
po, CR, tab, 200 mg	(*S*)-metoprolol	NM	739.36	512.79	1.44	42.03	28.11	1.50	Parker et al. 2011 [39]
po, tab, 100 mg	(*S*)-metoprolol	NM	280.28	191.91	1.46	87.63	76.15	1.15	Hemeryck et al. 2000 [15]
po, tab, 100 mg	(*S*)-metoprolol	AS=2.0	309.70	328.25	0.94	65.03	64.97	1.00	Huang et al. 1999 [17]
po, tab, 100 mg	(*S*)-metoprolol	AS=1.25	476.96	416.56	1.14	92.01	68.17	1.35	Huang et al. 1999 [17]
po, tab, 100 mg	(*S*)-metoprolol	AS=0.5	749.80	712.83	1.05	108.67	111.98	0.97	Huang et al. 1999 [17]
po, tab, 100 mg, bid	(*S*)-metoprolol	NM	602.11	446.50	1.35	86.88	81.12	1.07	Luzier et al. 1999 a[30]
po, tab, 100 mg, bid	(*S*)-metoprolol	NM	727.77	991.69	0.73	109.92	130.35	0.84	Luzier et al. 1999 b[31]
po, tab, 100 mg, bid	(*S*)-metoprolol	NM	541.62	581.77	1.29	93.65	79.62	1.34	Parker et al. 2011 [39]
po, CR, tab, 100 mg	(*S*)-metoprolol	NM	360.97	279.26	0.93	20.88	15.58	1.18	Parker et al. 2011 [39]
po, tab, 100 mg	(*S*)-metoprolol	AS=3.0	89.57	101.02	0.89	33.56	36.43	0.92	Seeringer et al. 2008 [46]
po, tab, 100 mg	(*S*)-metoprolol	AS=2.0	232.74	245.02	0.95	51.27	61.24	0.84	Seeringer et al. 2008 [46]
po, tab, 100 mg	(*S*)-metoprolol	AS=0.0	1552.77	1280.16	1.21	117.90	142.52	0.83	Seeringer et al. 2008 [46]
po, -, 100 mg	(*S*)-metoprolol	AS=1.5	452.28	580.03	0.78	95.42	97.80	0.98	Sharma et al. 2005 [47]
po, -, 100 mg	(*S*)-metoprolol	AS=0.0	2885.08	2387.00	1.21	186.00	232.43	0.80	Sharma et al. 2005 [47]
GMFE					1.21 (1.03–1.84)<br>32/32 with GMFE $\leq$ 2			1.19 (1.00–1.65)<br>32/32 with GMFE $\leq$ 2	
Overall GMFE (all four compounds)					1.27 (1.01–2.94)<br>72/75 with GMFE $\leq$ 2			1.23 (1.00–2.97)<br>64/66 with GMFE $\leq$ 2	

-: not available, AS: CYP2D6 activity score, AUC_{last}: AUC from the time of the first concentration measurement to the last time point of concentration measurement, bid: twice daily, CR: controlled release, GMFE: geometric mean fold error, NM: normal metabolizer, po: oral, Pred: predicted, Obs: observed, tab: tablet.

S2.6.7 Sensitivity analysis

Sensitivity of the final metoprolol model to single parameters (local sensitivity analysis) was calculated as the relative change of the $AUC_{0-24\,h}$ of a 100 mg single dose of metoprolol tartrate administered as tablet in the fasted state. Sensitivity analysis was carried out using a relative parameter perturbation of 1000% (variation range 10.0, maximum number of 9 steps). Parameters were included into the analysis if they were optimized (CYP2D6 k_{cat}, unspecific clearance, weibull shape and dissolution time (50% dissolved), intestinal permeability), if they were associated with optimized parameters (CYP2D6 K_m) or if they might have had a strong impact due to calculation methods used in the model (solubility, lipophilicity, fraction unbound).

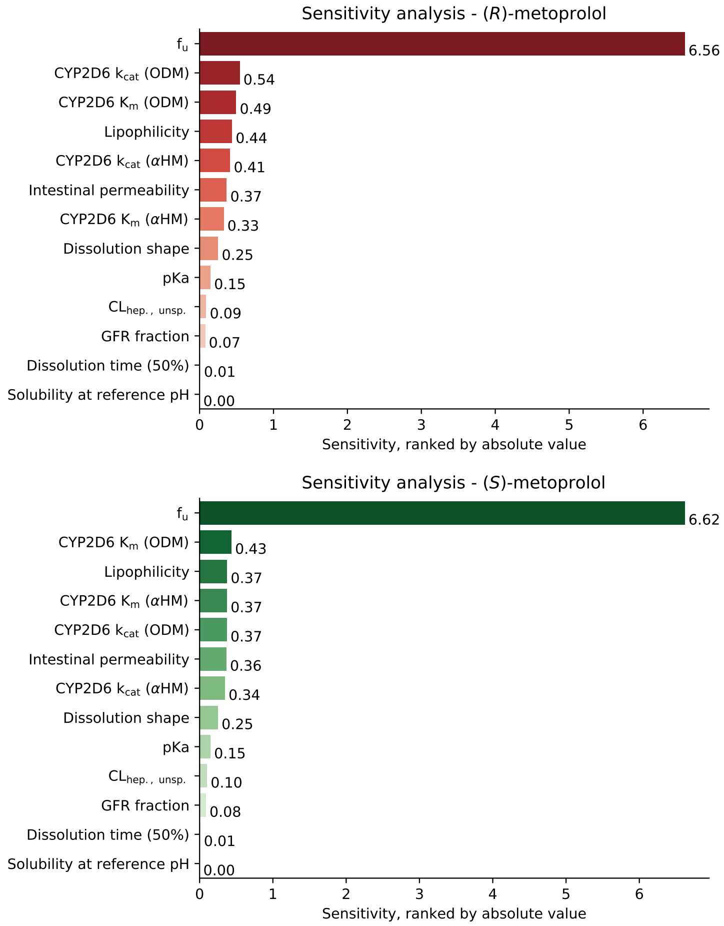

Figure S2.6.15: Sensitivity analysis of the (*R*)-metoprolol (upper panel) and (*S*)-metoprolol (lower panel) model. A sensitivity of +1.0 signifies that a 10% increase of the examined parameter value causes a 10% increase of the simulated $AUC_{0-24\,h}$. αHM: α-hydroxymetoprolol, CYP2D6: cytochrome P450 2D6, f_u: fraction unbound, GFR: glomerular filtration rate, k_{cat}: catalytic rate constant, K_m: Michaelis-Menten constant, ODM: *O*-desmethylmetoprolol.

S3 Metoprolol CYP2D6 DGI model

S3.1 Metoprolol k_{cat} values for the modeled activity scores

Table S3.1.1: $k_{cat,\ rel}$ values for the different CYP2D6 activity scores

	(*R*)-metoprolol		(*S*)-metoprolol		
Activity score	$k_{cat} \rightarrow \alpha$HM	$k_{cat} \rightarrow$ ODM	$k_{cat} \rightarrow \alpha$HM	$k_{cat} \rightarrow$ ODM	$k_{cat,\ rel}$
0	0.00 1/min	0.00 1/min	0.00 1/min	0.00 1/min	0%
0.5	1.65 1/min	2.70 1/min	1.82 1/min	2.27 1/min	19%
1.25	5.73 1/min	9.40 1/min	6.30 1/min	7.89 1/min	64%
1.5	6.38 1/min	10.48 1/min	7.03 1/min	8.81 1/min	72%
2	10.17 1/min	16.69 1/min	11.19 1/min	14.02 1/min	100%
3	19.03 1/min	31.22 1/min	20.93 1/min	26.23 1/min	213%

αHM: α-hydroxylation, k_{cat}: catalytic rate constant, $k_{cat,\ rel}$: k_{cat} relative to AS=2, ODM: *O*-demethylation

S3.2 Plasma profiles

S3.2.1 Semilogarithmic plots

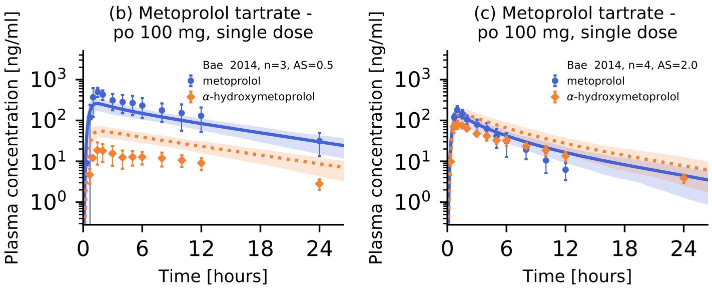

Figure S3.2.1: Metoprolol plasma concentrations of the modeled CYP2D6 drug-gene interaction. Model predictions of metoprolol and α-hydroxymetoprolol plasma concentration-time profiles of the CYP2D6 DGI study, compared to observed data [3] (semilogarithmic representation). Population predictions (n=100) are shown as lines with ribbons (arithmetic mean $\pm$ standard deviation (SD)), symbols represent the corresponding observed data $\pm$ SD. AS: activity score, oral (po): oral

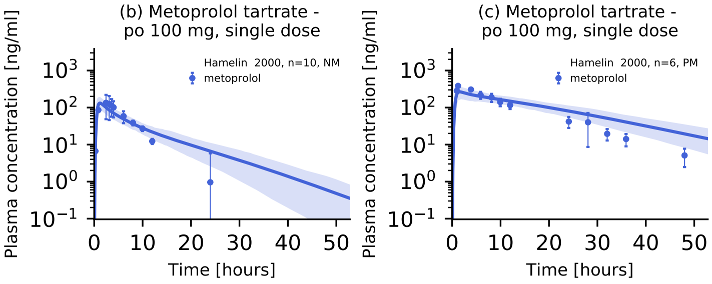

Figure S3.2.2: Metoprolol plasma concentrations of the modeled CYP2D6 drug-gene interaction. Model predictions of metoprolol plasma concentration-time profiles of the CYP2D6 DGI study, compared to observed data [14] (semilogarithmic representation). Population predictions (n=100) are shown as lines with ribbons (arithmetic mean $\pm$ standard deviation (SD)), symbols represent the corresponding observed data $\pm$ SD. NM: normal metabolizer, PM: poor metabolizer, po: oral

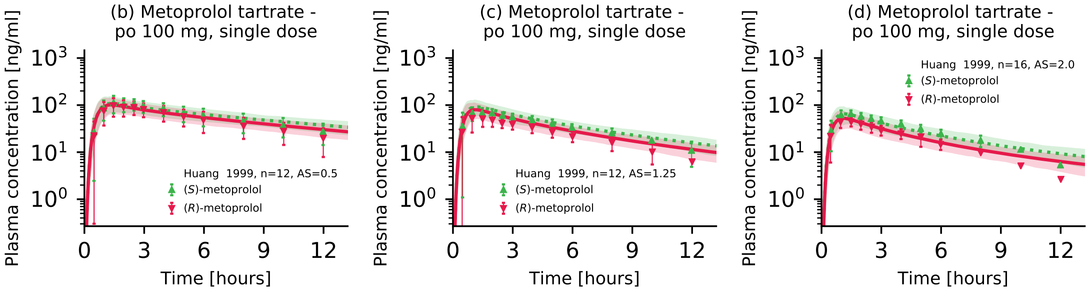

Figure S3.2.3: Metoprolol plasma concentrations of the modeled CYP2D6 drug-gene interaction. Model predictions of (*S*)-metoprolol and (*R*)-metoprolol plasma concentration-time profiles of the CYP2D6 DGI study, compared to observed data [17] (semilogarithmic representation). Population predictions (n=100) are shown as lines with ribbons (arithmetic mean ± standard deviation (SD)), symbols represent the corresponding observed data ± SD. AS: activity score, po: oral

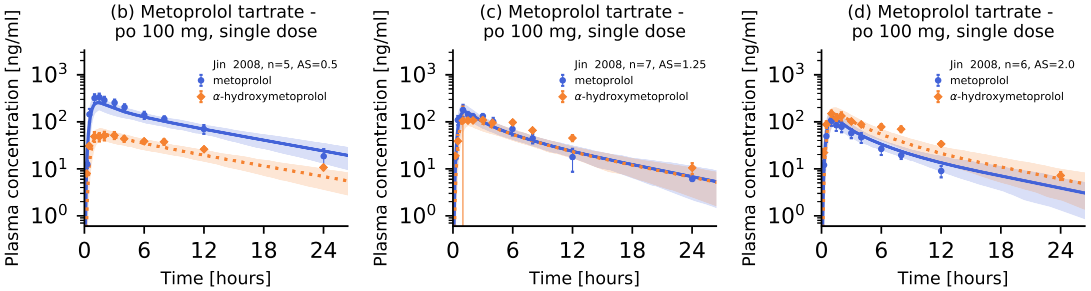

Figure S3.2.4: Metoprolol plasma concentrations of the modeled CYP2D6 drug-gene interaction. Model predictions of metoprolol and α-hydroxymetoprolol plasma concentration-time profiles of the CYP2D6 DGI study, compared to observed data [19] (semilogarithmic representation). Population predictions (n=100) are shown as lines with ribbons (arithmetic mean ± standard deviation (SD)), symbols represent the corresponding observed data ± SD. AS: activity score, po: oral

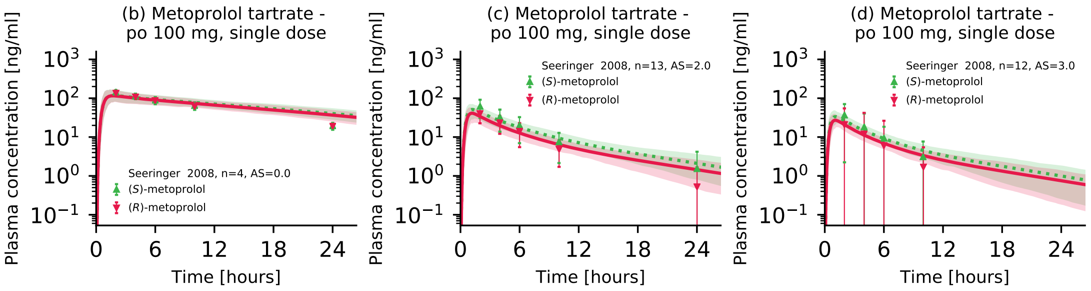

Figure S3.2.5: Metoprolol plasma concentrations of the modeled CYP2D6 drug-gene interaction. Model predictions of (*S*)-metoprolol and (*R*)-metoprolol plasma concentration-time profiles of the CYP2D6 DGI study, compared to observed data [46] (semilogarithmic representation). Population predictions (n=100) are shown as lines with ribbons (arithmetic mean ± standard deviation (SD)), symbols represent the corresponding observed data ± SD. AS: activity score, po: oral

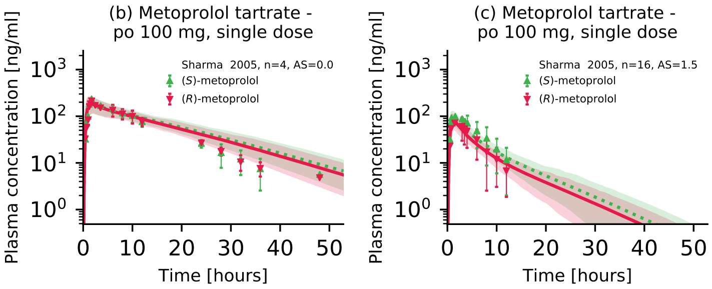

Figure S3.2.6: Metoprolol plasma concentrations of the modeled CYP2D6 drug-gene interaction. Model predictions of metoprolol, (*S*)-metoprolol and (*R*)-metoprolol plasma concentration-time profiles of the CYP2D6 DGI study, compared to observed data [47] (semilogarithmic representation). Population predictions (n=100) are shown as lines with ribbons (arithmetic mean ± standard deviation (SD)), symbols represent the corresponding observed data ± SD. AS: activity score, po: oral

S3.2.2 Linear plots

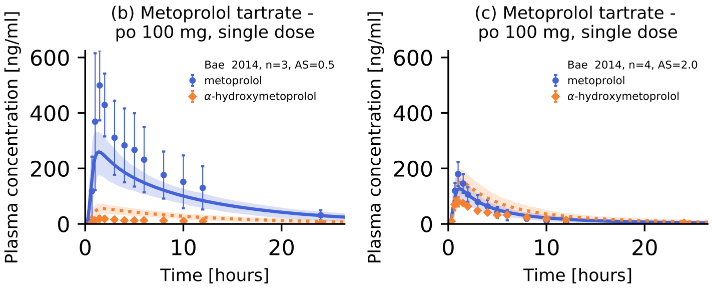

Figure S3.2.7: Metoprolol plasma concentrations of the modeled CYP2D6 drug-gene interaction. Model predictions of metoprolol and α-hydroxymetoprolol plasma concentration-time profiles of the CYP2D6 DGI study, compared to observed data [3] (linear representation). Population predictions (n=100) are shown as lines with ribbons (arithmetic mean $\pm$ standard deviation (SD)), symbols represent the corresponding observed data $\pm$ SD. AS: activity score, po: oral

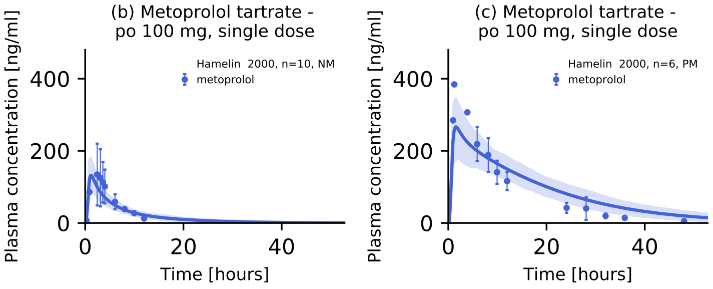

Figure S3.2.8: Metoprolol plasma concentrations of the modeled CYP2D6 drug-gene interaction. Model predictions of metoprolol plasma concentration-time profiles of the CYP2D6 DGI study, compared to observed data [14] (linear representation). Population predictions (n=100) are shown as lines with ribbons (arithmetic mean $\pm$ standard deviation (SD)), symbols represent the corresponding observed data $\pm$ SD. NM: normal metabolizer, PM: poor metabolizer, po: oral

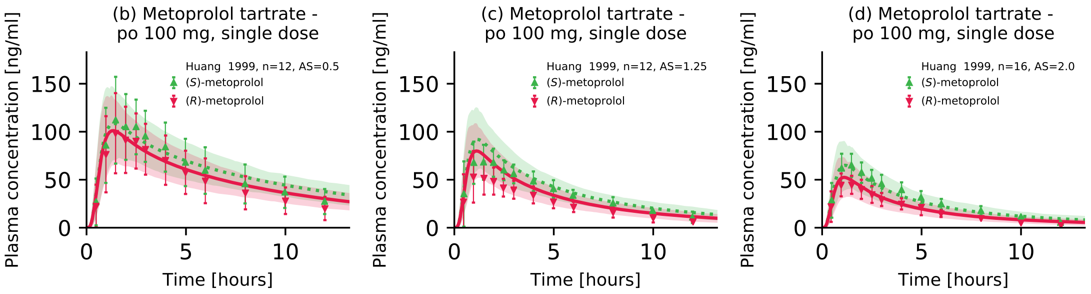

Figure S3.2.9: Metoprolol plasma concentrations of the modeled CYP2D6 drug-gene interaction. Model predictions of (*S*)-metoprolol and (*R*)-metoprolol plasma concentration-time profiles of the CYP2D6 DGI study, compared to observed data [17] (linear representation). Population predictions (n=100) are shown as lines with ribbons (arithmetic mean ± standard deviation (SD)), symbols represent the corresponding observed data ± SD. AS: activity score, po: oral

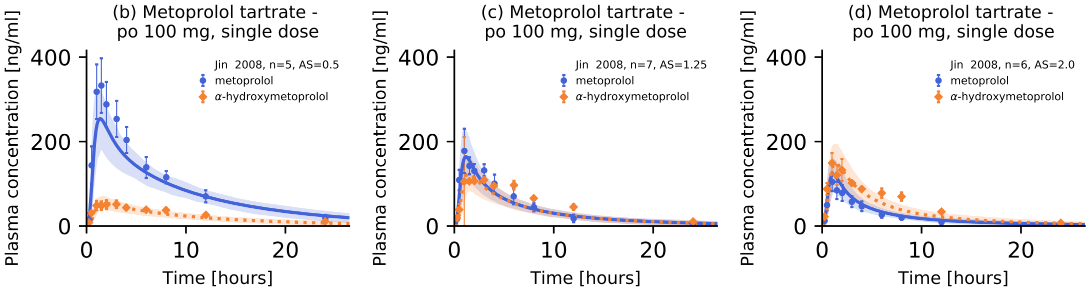

Figure S3.2.10: Metoprolol plasma concentrations of the modeled CYP2D6 drug-gene interaction. Model predictions of metoprolol and α-hydroxymetoprolol plasma concentration-time profiles of the CYP2D6 DGI study, compared to observed data [19] (linear representation). Population predictions (n=100) are shown as lines with ribbons (arithmetic mean ± standard deviation (SD)), symbols represent the corresponding observed data ± SD. AS: activity score, po: oral

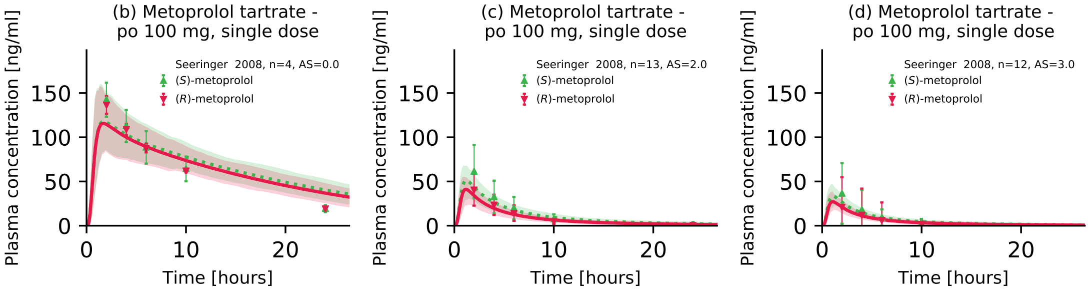

Figure S3.2.11: Metoprolol plasma concentrations of the modeled CYP2D6 drug-gene interaction. Model predictions of (*S*)-metoprolol and (*R*)-metoprolol plasma concentration-time profiles of the CYP2D6 DGI study, compared to observed data [46] (linear representation). Population predictions (n=100) are shown as lines with ribbons (arithmetic mean ± standard deviation (SD)), symbols represent the corresponding observed data ± SD. AS: activity score, po: oral

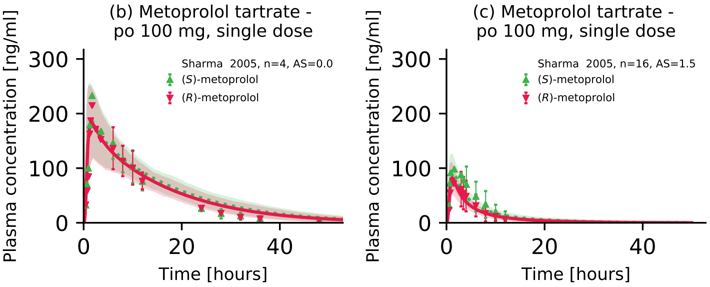

Figure S3.2.12: Metoprolol plasma concentrations of the modeled CYP2D6 drug-gene interaction. Model predictions of metoprolol, (*S*)-metoprolol and (*R*)-metoprolol plasma concentration-time profiles of the CYP2D6 DGI study, compared to observed data [47] (linear representation). Population predictions (n=100) are shown as lines with ribbons (arithmetic mean ± standard deviation (SD)), symbols represent the corresponding observed data ± SD. AS: activity score, po: oral

S3.3 Model evaluation

S3.3.1 Metoprolol CYP2D6 DGI AUC_{last} and C_{max} ratio plots

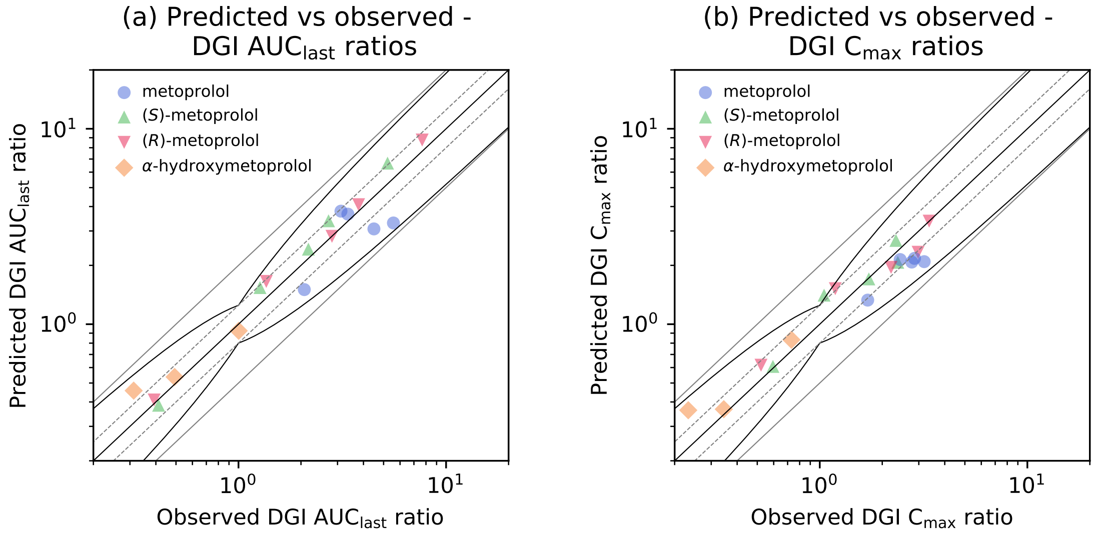

Figure S3.3.13: Predicted versus observed metoprolol DGI ratios. Comparison of predicted versus observed AUC_{last} ratios (a) and C_{max} ratios (b) for metoprolol CYP2D6 DGI-studies. The straight black line indicates the line of identity, curved black lines show prediction success limits proposed by Guest et al. including 1.25-fold variability [13]. Solid light grey lines indicate 2-fold deviation, dashed light grey lines show 1.25-fold deviation. AUC_{last}: AUC from the time of the first concentration measurement to the last time point of concentration measurement, C_{max}: peak plasma concentration, DGI: drug-gene interaction

S3.3.2 Geometric mean fold error of predicted metoprolol DGI AUC_{last} and C_{max} ratios

Table S3.3.2: Geometric mean fold error of predicted metoprolol DGI AUC_{last} and C_{max} ratios

Molecule	Dosing	Activity score	*CYP2D6* Genotype	CYP2D6 Phenotype	DGI AUC_{last} ratio			DGI C_{max} ratio			Reference
					Pred	Obs	Pred/Obs	Pred	Obs	Pred/Obs	
α-hydroxymetoprolol	100 mg, tab, sd	0.5	**10/*10*	IM	0.46	0.31	1.46	0.36	0.23	1.56	Bae et al. 2014 [3]
α-hydroxymetoprolol	100 mg, tab, sd	1.25	**1/*10*	NM	0.93	1.00	0.92	0.83	0.73	1.14	Jin et al. 2008 [19]
α-hydroxymetoprolol	100 mg, tab, sd	0.5	**10/*10*	IM	0.54	0.49	1.09	0.37	0.34	1.06	Jin et al. 2008 [19]
metoprolol	100 mg, tab, sd	0.5	**10/*10*	IM	3.30	5.58	1.69	2.08	2.77	0.75	Bae et al. 2014 [3]
metoprolol	100 mg, tab, sd	-	-	PM	3.66	3.36	1.09	2.17	2.86	0.76	Hamelin et al. 2000 [14]
metoprolol	100 mg, tab, sd	1.25	**1/*10*	NM	1.50	2.08	0.72	1.33	1.70	0.78	Jin et al. 2008 [19]
metoprolol	100 mg, tab, sd	0.5	**10/*10*	IM	3.07	4.49	0.68	2.09	3.18	0.66	Jin et al. 2008 [19]
metoprolol	100 mg, -, sd	0	†	PM	3.79	3.12	1.22	2.14	2.44	0.88	Sharma et al. 2005 [47]
(*R*)-metoprolol	100 mg, tab, sd	1.25	**1/*10*	NM	1.65	1.36	1.22	1.52	1.19	1.28	Huang et al. 1999 [17]
(*R*)-metoprolol	100 mg, tab, sd	0.5	**10/*10*	IM	2.82	2.82	1.00	1.95	2.21	0.89	Huang et al. 1999 [17]
(*R*)-metoprolol	100 mg, tab, sd	3	†	UM	0.41	0.39	1.04	0.62	0.52	1.18	Seeringer et al. 2008 [46]
(*R*)-metoprolol	100 mg, tab, sd	0	†	PM	8.76	7.67	1.14	3.37	3.36	1.00	Seeringer et al. 2008 [46]
(*R*)-metoprolol	100 mg, -, sd	0	†	PM	4.09	3.79	1.08	2.33	2.98	0.78	Sharma et al. 2005 [47]
(*S*)-metoprolol	100 mg, tab, sd	1.25	**1/*10*	NM	1.54	1.27	1.21	1.41	1.05	1.34	Huang et al. 1999 [17]
(*S*)-metoprolol	100 mg, tab, sd	0.5	**10/*10*	IM	2.42	2.17	1.11	1.71	1.72	0.99	Huang et al. 1999 [17]
(*S*)-metoprolol	100 mg, tab, sd	3	†	UM	0.38	0.41	0.93	0.61	0.59	1.02	Seeringer et al. 2008 [46]
(*S*)-metoprolol	100 mg, tab, sd	0	†	PM	6.67	5.22	1.28	2.68	2.33	1.15	Seeringer et al. 2008 [46]
(*S*)-metoprolol	100 mg, -, sd	0	†	PM	3.38	2.72	1.25	2.07	2.38	0.87	Sharma et al. 2005 [47]
Overall GMFE							1.21 (1.00–1.69)<br>18/18 with GMFE $\leq$ 2			1.21 (1.00–1.56)<br>18/18 with GMFE $\leq$ 2	
Ratios within the limits of *Guest et al.* [13] (including 1.25-fold deviation)							18/18			17/18	

-: not available, †: mixed genotype (given in publication), AUC_{last}: AUC from the time of the first concentration measurement to the last time point of concentration measurement, C_{max}: peak plasma concentration, CYP2D6: cytochrome P450 2D6, IM: intermediate metabolizer, NM: normal metabolizer, Obs: observed, PM: poor metabolizer, Pred: predicted, sd: single dose, tab: tablet, UM: ultrarapid metabolizer

S4 System-dependent parameters

Details on the implementation of CYP2D6 are summarized in Table S4.0.1.

Table S4.0.1: System-dependent parameters

Enyzme	Reference concentration		Localization	Half-life	
	Mean[a]	Relative expression[b]		Liver [h]	Intestine [h]
CYP2D6	0.40 [45]	RT-PCR [38]	intracellular	51 [38]	23 [38]
EHC fraction: Fraction of biliary secreted compound directly entering the duodenum = 1					

[a]: µmol protein/l in the tissue of highest expression
[b]: In the different organs (PK-Sim expression database profile)
CYP2D6: cytochrome P450 2D6, EHC: enterohepatic circulation, RT-PCR: reverse transcription-polymerase chain reaction profile

S5 Abbreviations

ρ_i ith observed plasma AUC_{last} or C_{max} value

$\hat{\rho}_i$ ith predicted plasma AUC_{last} or C_{max} value

ADME Absorption, distribution, metabolism and excretion

AS CYP2D6 activity score

AUC Area under the plasma concentration-time curve

AUC_{last} AUC from the time of the first concentration measurement to the last time point of concentration measurement

BA Bioavailability

bid Twice daily

BCS Biopharmaceutics Classification System

c_i ith observed plasma concentration

$\hat{c}_i$ ith predicted plasma concentration

CL Clearance

$CL_{hep, unsp.}$ Unspecific hepatic clearance

C_{max} Peak plasma concentration

CR Controlled release

CYP2B6 Cytochrome P450 2B6

CYP2C9 Cytochrome P450 2C9

CYP2D6 Cytochrome P450 2D6

CYP3A4 Cytochrome P450 3A4

DDI Drug-drug interaction

DGI Drug-gene interaction

EHC Enterohepatic circulation

FDA U. S. Food and Drug Administration

$\mathbf{f_u}$ Fraction unbound

g- Genotyped

GFR Glomerular filtration rate

GMFE Geometric mean fold error

ICRP International Commission on Radiological Protection

IM Intermediate metabolizer

inf Infusion

iv Intravenous

$\mathbf{k_{cat}}$ Catalytic rate constant

$k_{cat,\ rel}$ Catalytic rate constant relative to AS=2

K_m Michaelis-Menten constant

MRD Mean relative deviation

MW Molecular weight

NHANES Third National Health and Nutrition Examination Survey

NM Normal metabolizer

NR Normal release

p- Phenotyped

PBPK Physiologically based pharmacokinetic

pKa Acid dissociation constant

PM Poor metabolizer

po Oral

QSAR Quantitative structure-activity relationship

sd Single dose

sol Oral solution

tab Tablet

UM Ultrarapid metabolizer

v_{max} Maximum reaction velocity

Bibliography

1. Austin, R. P., Barton, P., Cockroft, S. L., Wenlock, M. C. & Riley, R. J. The influence of nonspecific microsomal binding on apparent intrinsic clearance, and its prediction from physicochemical properties. *Drug Metabolism and Disposition* **30,** 1497–1503 (2002).
2. Avdeef, A. & Berger, C. M. pH-metric solubility.: 3. Dissolution titration template method for solubility determination. *European Journal of Pharmaceutical Sciences* **14,** 281–291 (2001).
3. Bae, S. H., Lee, J. K., Cho, D.-Y. & Bae, S. K. Simultaneous determination of metoprolol and its metabolites, α-hydroxymetoprolol and O-desmethylmetoprolol, in human plasma by liquid chromatography with tandem mass spectrometry: Application to the pharmacokinetics of metoprolol associated with CYP2D6 g. *Journal of separation science* **37,** 1256–64 (June 2014).
4. Bennett, P. N., John, V. A. & Whitmarsh, V. B. Effect of rifampicin on metoprolol and antipyrine kinetics. *British journal of clinical pharmacology* **13,** 387–91 (Mar. 1982).
5. Berger, B., Bachmann, F., Duthaler, U., Krähenbühl, S. & Haschke, M. Cytochrome P450 enzymes involved in metoprolol metabolism and use of metoprolol as a CYP2D6 phenotyping probe drug. *Frontiers in Pharmacology* **9,** 1–11 (2018).
6. Boldhane, S. & Kuchekar, B. Development and optimization of metoprolol succinate gastroretentive drug delivery system. *Acta Pharmaceutica* **60,** 415–425 (2010).
7. Bozkurt, A., Başçi, N. B., Işimer, A., Sayal, A. & Kayaalp, S. O. Metabolic ratios of four probes of CYP2D6 in Turkish subjects: A cross-over study. *European Journal of Drug Metabolism and Pharmacokinetics* **21,** 309–314 (1996).
8. Cerqueira, P. M. *et al.* Enantioselectivity in the steady-state pharmacokinetics of metoprolol in hypertensive patients. *Chirality* **11,** 591–597.
9. Chellingsworth, M. C., Laugher, S., Akhlaghi, S., Jack, D. B. & Kendall, M. J. The effects of ranitidine and cimetidine on the pharmacokinetics and pharmacodynamics of metoprolol. *Alimentary pharmacology & therapeutics* **2,** 521–7 (Dec. 1988).
10. ClinCalc LLC. *ClinCalc DrugStats Database* `https://clincalc.com/Drugstats/` (2020).
11. Damy, T., Pousset, F., Caplain, H., Hulot, J.-S. S. & Lechat, P. Pharmacokinetic and pharmacodynamic interactions between metoprolol and dronedarone in extensive and poor CYP2D6 metabolizers healthy subjects. *Fundamental and Clinical Pharmacology* **18,** 113–123 (Feb. 2004).

12. Godbillon, J. *et al.* Investigation of drug absorption from the gastrointestinal tract of man. III. Metoprolol in the colon. *British journal of clinical pharmacology* **19 Suppl 2,** 113S–118S (Apr. 1985).

13. Guest, E. J., Aarons, L., Houston, J. B., Rostami-Hodjegan, A. & Galetin, A. Critique of the two-fold measure of prediction success for ratios: application for the assessment of drug-drug interactions. *Drug metabolism and disposition: the biological fate of chemicals* **39,** 170–3 (Feb. 2011).

14. Hamelin, B. A. *et al.* Significant interaction between the nonprescription antihistamine diphenhydramine and the CYP2D6 substrate metoprolol in healthy men with high or low CYP2D6 activity. *Clinical pharmacology and therapeutics* **67,** 466–77 (May 2000).

15. Hemeryck, A., Lefebvre, R. A., De Vriendt, C. & Belpaire, F. M. Paroxetine affects metoprolol pharmacokinetics and pharmacodynamics in healthy volunteers. *Clinical pharmacology and therapeutics* **67,** 283–91 (Mar. 2000).

16. Houtzagers, J. J., Streurman, O. & Regårdh, C. G. The effect of pretreatment with cimetidine on the bioavailability and disposition of atenolol and metoprolol. *British journal of clinical pharmacology* **14,** 67–72 (July 1982).

17. Huang, J., Chuang, S. K., Cheng, C. L. & Lai, M. L. Pharmacokinetics of metoprolol enantiomers in Chinese subjects of major CYP2D6 genotypes. *Clinical pharmacology and therapeutics* **65,** 402–7 (Apr. 1999).

18. Jack, D. B. *et al.* The effect of hydralazine on the pharmacokinetics of three different beta adrenoceptor antagonists: Metoprolol, nadolol, and acebutolol. *Biopharmaceutics & drug disposition* **3,** 47–54.

19. Jin, S. K. *et al.* Influence of CYP2D6*10 on the pharmacokinetics of metoprolol in healthy Korean volunteers. *Journal of clinical pharmacy and therapeutics* **33,** 567–73.

20. Johnson, J. A. & Burlew, B. S. Metoprolol metabolism via cytochrome P4502D6 in ethnic populations. *Drug metabolism and disposition: the biological fate of chemicals* **24,** 350–5 (Mar. 1996).

21. Johnson, J. A. & Burlew, B. S. Metoprolol metabolism via cytochrome P4502D6 in ethnic populations. *Drug metabolism and disposition: the biological fate of chemicals* **24,** 350–5 (Mar. 1996).

22. Johnsson, G., Regärdh, C.-G. & Sölvell, L. Combined pharmacokinetic and pharmacodynamic studies in man of the adrenergic β1-receptor antagonist metoprolol. *Acta Pharmacologica et Toxicologica* **36,** 31–44 (Mar. 1975).

23. Kelly, J. G., Salem, S. A., Kinney, C. D., Shanks, R. G. & McDevitt, D. G. Effects of ranitidine on the disposition of metoprolol. *British Journal of Clinical Pharmacology* **19,** 219–224 (Feb. 1985).

24. Kim, S. *et al.* PubChem 2019 update: Improved access to chemical data. *Nucleic Acids Research* **47,** D1102–D1109 (2019).

25. Kirchheiner, J. *et al.* Impact of the ultrarapid metabolizer genotype of cytochrome P450 2D6 on metoprolol pharmacokinetics and pharmacodynamics. *Clinical pharmacology and therapeutics* **76,** 302–12 (Oct. 2004).

26. Krauwinkel, W. *et al.* The effect of mirabegron, a potent and selective $\beta 3$-adrenoceptor agonist, on the pharmacokinetics of CYP2D6 substrates desipramine and metoprolol. *European journal of drug metabolism and pharmacokinetics* **39,** 43–52 (Mar. 2014).

27. Krösser, S. *et al.* Investigation of sarizotan's impact on the pharmacokinetics of probe drugs for major cytochrome P450 isoenzymes: a combined cocktail trial. *European Journal of Clinical Pharmacology* **62,** 277–84.

28. Langenbucher, F. Linearization of dissolution rate curves by the Weibull distribution. *The Journal of pharmacy and pharmacology* **24,** 979–81. `http://www.ncbi.nlm.nih.gov/pubmed/4146531` (Dec. 1972).

29. Lennard, M. S., Tucker, G. T., Silas, J. H. & Woods, H. F. Debrisoquine polymorphism and the metabolism and action of metoprolol, timolol, propranolol and atenolol. *Xenobiotica* **16,** 435–447.

30. Luzier, A. B. *et al.* Gender-related effects on metoprolol pharmacokinetics and pharmacodynamics in healthy volunteers. *Clinical pharmacology and therapeutics* **66,** 594–601 (Dec. 1999).

31. Luzier, A. B. *et al.* Gender-related effects on metoprolol pharmacokinetics and pharmacodynamics in healthy volunteers. *Clinical pharmacology and therapeutics* **66,** 594–601 (Dec. 1999).

32. Mateus, A., Matsson, P. & Artursson, P. A high-throughput cell-based method to predict the unbound drug fraction in the brain. *Journal of Medicinal Chemistry* **57,** 3005–3010.

33. Mautz, D. S., Nelson, W. L. & Shen, D. D. Regioselective and stereoselective oxidation of metoprolol and bufuralol catalyzed by microsomes containing cDNA-expressed human P4502D6. *Drug metabolism and disposition: the biological fate of chemicals* **23,** 513–7 (Apr. 1995).

34. Nathanson, J. A. Stereospecificity of beta adrenergic antagonists: R-enantiomers show increased selectivity for beta-2 receptors in ciliary process. *The Journal of pharmacology and experimental therapeutics* **245,** 94–101 (Apr. 1988).

35. National Center for Health Statistics Hyattsville MD 20782. *Third National Health and Nutrition Examination Survey, (NHANES III)* tech. rep. (1997).

36. Novartis Pharmaceuticals Corporation. *Lopressor® tablet and injection - Prescribing information* `https://www.accessdata.fda.gov/drugsatfda%7B%5C_%7Ddocs/label/2008/017963s062,018704s021lbl.pdf` (2020).

37. Open Systems Pharmacology Suite Community. Open Systems Pharmacology Suite Manual, Version 7.4. `https://github.com/Open-Systems-Pharmacology/OSPSuite.Documentation/blob/master/Open%20Systems%20Pharmacology%20Suite.pdf` (2018).

38. Open Systems Pharmacology Suite Community. PK-Sim® Ontogeny Database Documentation, Version 7.3. `https://github.com/Open-Systems-Pharmacology/OSPSuite.Documentation/blob/master/PK-Sim%20Ontogeny%20Database%20Version%207.3.pdf` (2018).
39. Parker, R. B. & Soberman, J. E. Effects of paroxetine on the pharmacokinetics and pharmacodynamics of immediate-release and extended-release metoprolol. *Pharmacotherapy* **31,** 630–641 (2011).
40. Plosker, G. L. & Clissold, S. P. Controlled Release Metoprolol Formulations. *Drugs* **43,** 382–414 (Mar. 1992).
41. Regårdh, C. G., Borg, K. O., Johansson, R., Johnsson, G. & Palmer, L. Pharmacokinetic studies on the selective beta1-receptor antagonist metoprolol in man. *Journal of pharmacokinetics and biopharmaceutics* **2,** 347–64 (Aug. 1974).
42. Regårdh, C. G. & Johnsson, G. Clinical Pharmacokinetics of Metoprolol. *Clinical Pharmacokinetics* **5,** 557–569 (1980).
43. Rodgers, T., Leahy, D. & Rowland, M. Physiologically based pharmacokinetic modeling 1: predicting the tissue distribution of moderate-to-strong bases. *Journal of pharmaceutical sciences* **94,** 1259–76. `http://www.ncbi.nlm.nih.gov/pubmed/15858854` (June 2005).
44. Rodgers, T. & Rowland, M. Physiologically based pharmacokinetic modelling 2: predicting the tissue distribution of acids, very weak bases, neutrals and zwitterions. *Journal of pharmaceutical sciences* **95,** 1238–57. arXiv: `z0024` (June 2006).
45. Rodrigues, A. D. Integrated cytochrome P450 reaction phenotyping: attempting to bridge the gap between cDNA-expressed cytochromes P450 and native human liver microsomes. *Biochemical pharmacology* **57,** 465–80 (1999).
46. Seeringer, A., Brockmöller, J., Bauer, S. & Kirchheiner, J. Enantiospecific pharmacokinetics of metoprolol in CYP2D6 ultra-rapid metabolizers and correlation with exercise-induced heart rate. *European journal of clinical pharmacology* **64,** 883–8 (Sept. 2008).
47. Sharma, A. *et al.* Modulation of Metoprolol Pharmacokinetics and Hemodynamics by Diphenhydramine Coadministration during Exercise Testing in Healthy Premenopausal Women. *Journal of Pharmacology and Experimental Therapeutics* **313,** 1172–1181 (June 2005).
48. Stout, S. M. *et al.* Influence of metoprolol dosage release formulation on the pharmacokinetic drug interaction with paroxetine. *Journal of clinical pharmacology* **51,** 389–96 (Mar. 2011).
49. Tanaka, G. & Kawamura, H. Anatomical and physiological characteristics for Asian reference man: male and female of different ages: Tanaka model. Division of Radioecology, National Institute of Radiological Sciences. Hitachinaka 311-12 Japan. NIRS-M-115 (1996).

50. Thelen, K. *et al.* Evolution of a detailed physiological model to simulate the gastrointestinal transit and absorption process in humans, Part 1: Oral solutions. *Journal of Pharmaceutical Sciences* **100,** 5324–5345 (Dec. 2011).
51. U.S. Food and Drug Administration. *Drug Development and Drug Interactions: Table of Substrates, Inhibitors and Inducers | FDA* `https://www.fda.gov/drugs/drug-interactions-labeling/drug-development-and-drug-interactions-table-substrates-inhibitors-and-inducers` (2020).
52. Valentin, J. Basic anatomical and physiological data for use in radiological protection: reference values. A report of age- and gender-related differences in the anatomical and physiological characteristics of reference individuals. ICRP Publication 89. *Annals of the ICRP* **32,** 5–265 (2002).
53. Watanabe, R. *et al.* Predicting Fraction Unbound in Human Plasma from Chemical Structure: Improved Accuracy in the Low Value Ranges. *Molecular Pharmaceutics* **15,** 5302–5311 (2018).
54. Werner, U. *et al.* Celecoxib inhibits metabolism of cytochrome P450 2D6 substrate metoprolol in humans. *Clinical pharmacology and therapeutics* **74,** 130–137.
55. Wishart, D. S. *et al.* HMDB 4.0: the human metabolome database for 2018. *Nucleic Acids Research* **46,** D608–D617 (Jan. 2018).
56. Wojtyniak, J. G., Britz, H., Selzer, D., Schwab, M. & Lehr, T. Data Digitizing: Accurate and Precise Data Extraction for Quantitative Systems Pharmacology and Physiologically-Based Pharmacokinetic Modeling. *CPT: Pharmacometrics and Systems Pharmacology* **9,** 322–331 (2020).
57. Zhao, Y. *et al.* High-throughput logP measurement using parallel liquid chromatography/ultraviolet/mass spectrometry and sample-pooling. *Rapid communications in mass spectrometry : RCM* **16,** 1548–55 (2002).
